# Climate‐driven shifts in leaf senescence are greater for boreal species than temperate species in the Acadian Forest region in contrast to leaf emergence shifts

**DOI:** 10.1002/ece3.10362

**Published:** 2023-07-31

**Authors:** Lynsay Spafford, Andrew MacDougall, James Steenberg

**Affiliations:** ^1^ Climate and Environment Saint Francis Xavier University Antigonish Nova Scotia Canada; ^2^ Environmental Sciences Memorial University St. John's Newfoundland and Labrador Canada; ^3^ Nova Scotia Department of Natural Resources and Renewables Truro Nova Scotia Canada

**Keywords:** Acadian Forest, boreal, climate change, leaf phenology, process modelling, temperate

## Abstract

The Acadian Forest Region is a temperate‐boreal transitional zone in eastern North America which provides a unique opportunity for understanding the potential effects of climate change on both forest types. Leaf phenology, the timing of leaf life cycle changes, is an important indicator of the biological effects of climate change, which can be observed with stationary timelapse cameras known as phenocams. Using four growing seasons of observations for the species *Acer rubrum* (red maple), *Betula papyrifera* (paper/white birch) and *Abies balsamea* (balsam fir) from the Acadian Phenocam Network as well as multiple growing season observations from the North American PhenoCam Network we parameterized eight leaf emergence and six leaf senescence models for each species which span a range in process and driver representation. With climate models from the Fifth Phase of the Coupled Model Intercomparison Project (CMIP5) we simulated future leaf emergence, senescence and season length (senescence minus emergence) for these species at sites within the Acadian Phenocam Network. Model performances were similar across models and leaf emergence model RMSE ranged from about 1 to 2 weeks across species and models, while leaf senescence model RMSE ranged from about 2 to 4 weeks. The simulations suggest that by the late 21st century, leaf senescence may become continuously delayed for boreal species like *Betula papyrifera* and *Abies balsamea*, though remain relatively stable for temperate species like *Acer rubrum*. In contrast, the projected advancement in leaf emergence was similar across boreal and temperate species. This has important implications for carbon uptake, nutrient resorption, ecology and ecotourism for the Acadian Forest Region. More work is needed to improve predictions of leaf phenology for the Acadian Forest Region, especially with respect to senescence. Phenocams have the potential to rapidly advance process‐based model development and predictions of leaf phenology in the context of climate change.

## Graphical abstract

By the end of the 21st century under high emissions, leaf senescence and the length of the leafing period for temperate species such as red maple (*Acer rubrum*) may diverge from boreal tree species in the Acadian Forest Region. Phenocams are powerful monitoring tools that aid in developing robust and widely applicable process‐based models of leaf phenology, allowing us to explore future patterns in leaf phenology.

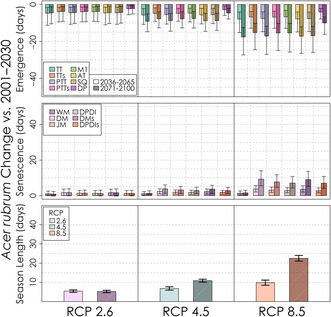

## 1 INTRODUCTION

Phenology, the timing of recurrent biological events, is influenced by climate and therefore an important indicator of the biological effects of climate change. To optimize growing season length and reproduction potential while avoiding exposure of vulnerable tissues to adverse conditions, plants undergo annual changes that are timed relative to environmental cues such as temperature and daylength (Vitasse et al., 2013). In the late growing season following budset, hormones from distal buds and leaves suppress bud development in what is known as paradormancy (Cline & Deppong, 1999). Following this phase, plants enter a state known as endodormancy or dormancy from autumn to winter, in which internal mechanisms within the bud limit bud cell growth. After sufficient exposure to chilling temperatures, plants enter ecodormancy or quiescence in which suboptimal growing conditions limit cell growth. Following sufficient exposure to warm temperatures, known as ‘forcing’, and sufficient daylength, ecodormancy release is observed as bud burst in which new leaves become visible (Delpierre et al., 2016). Later in the growing season, plants undergo leaf senescence and dormancy induction as daylength is shortened and temperatures become cooler (Beil et al., 2021; Caffarra et al., 2011).

Climate change is altering the timing of plant phenological events through changes in seasonal temperature and moisture regimes (Cleland et al., 2007; Kunkel et al., 2004; Piao et al., 2019; Scheifinger et al., 2003). Recent warming has generally led to earlier leaf emergence and delayed leaf senescence for most mid to high‐latitude tree species, culminating in an extension of the growing season (Estiarte & Peñuelas, 2015; Peñuelas & Filella, 2009; Polgar & Primack, 2011). Changes in leaf phenology have important implications for a range of processes on various spatiotemporal scales, including carbon cycling, water cycling, ecological interactions, susceptibility to unfavourable growing conditions or events and long‐term biogeographical range shifts (Chuine & Régnière, 2017; Cleland et al., 2007; Kharouba et al., 2018; Meier et al., 2021; Morin et al., 2009; Pureswaran et al., 2019; Renner & Zohner, 2018; Spafford et al., 2023). Consequently, characterizing and predicting future changes in leaf phenology is important for environmental and natural resource planning and climate change adaptation. Predicting future patterns in leaf phenology with increasing surface temperatures is challenging however due to a limited understandings of drivers and evolved cues, especially for leaf senescence (Chen et al., 2019; Delpierre et al., 2016; Gallinat et al., 2015; Keenan & Richardson, 2015; Piao et al., 2019).

Process‐based modelling of leaf phenology can provide insight into the species‐specific responses of leaf phenology to climate change and aid in predicting future leaf phenology patterns. Previous efforts have provided insight into potential future responses to climate change. Examples include shortened leaf colouration periods in autumn due to warming, heat stress or moisture stress (Xie, Wang, et al., 2018; Zohner & Renner, 2019), as well as non‐linear leaf emergence responses to further warming due to the constraining influence of photoperiod and chilling controls (Chen et al., 2019; Moon et al., 2021). Studies have also found evidence for additional nuanced controls of leaf phenology, such as bud albedo, interdependence between spring and autumn phenology, carbon uptake capacity limitation, response to biomass loss, variable sensitivity to drivers and others (Keenan & Richardson, 2015; Lang et al., 2019; Piao et al., 2019; Vitasse et al., 2021). Local‐scale experimental studies have developed valuable insights for process‐based modelling, though Wolkovich et al. (2012) reported that experimental studies may considerably underestimate phenological responses to warming relative to long‐term observations. Relatively few studies have yet explored species‐specific process‐based modelling employing observations over large regions to examine how broad controls in leaf phenology differ among species in natural contexts (Cook et al., 2012), as well as potential responses to future climate warming.

**FIGURE 1:**
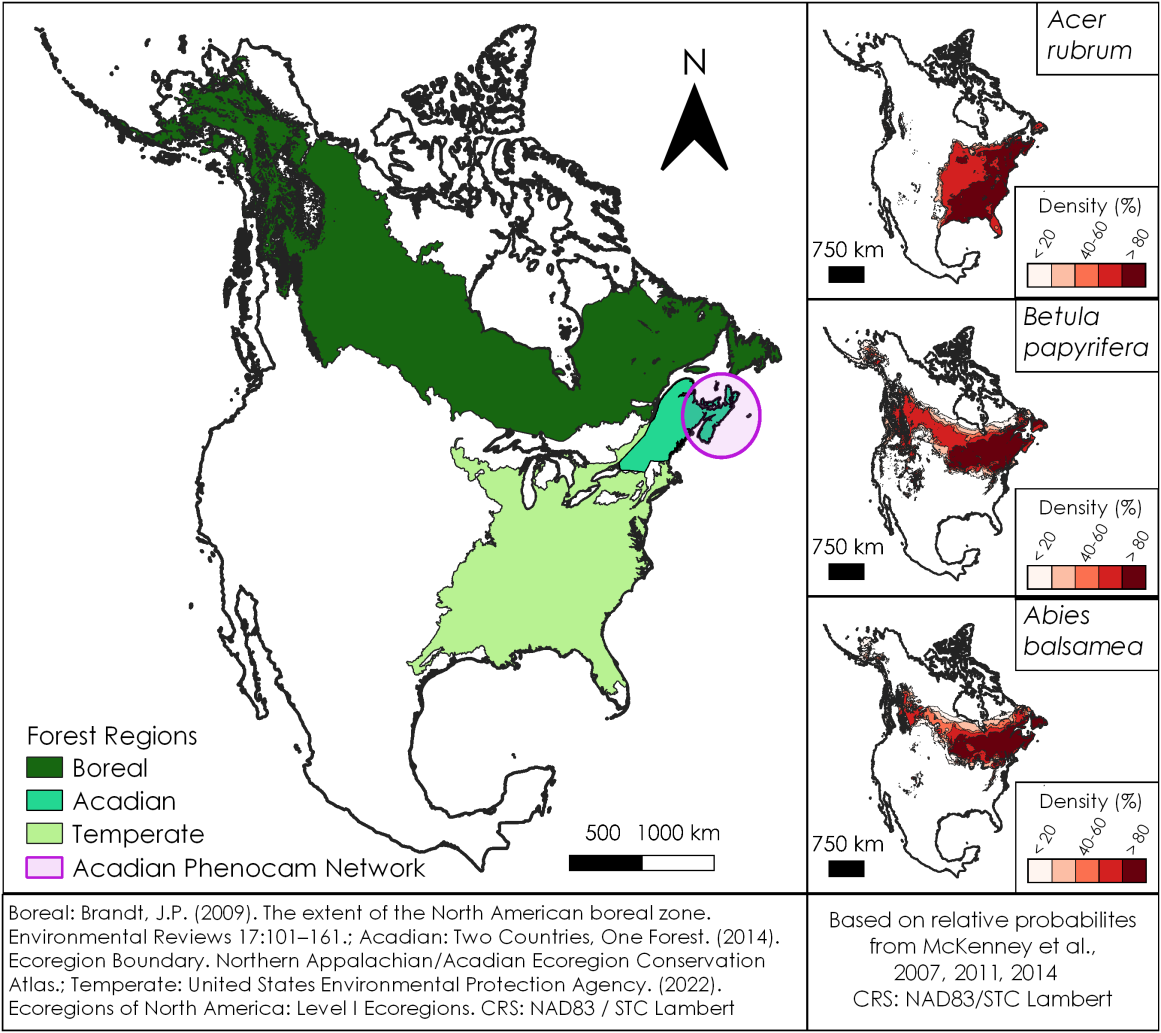
The distribution of the Boreal, Acadian and Temperate Forest Regions within North America (left) and the distribution of *Acer rubrum*, *Betula papyrifera* and *Abies balsamea* within North America (right). The ‘Acadian Forest Region’ depiction is based on forest composition and stand characteristics and is distinct from more detailed ecozone and ecosite classifications which incorporate a greater variety of environmental variables (Neily et al., 2013). The precise extent of the Acadian Forest Region differs among sources (Rowe, 1972; Two Countries One Forest, 2014).

While databases of leaf phenology observations are now globally extensive, there has been sparse in‐situ coverage of the Acadian Forest Region, especially for the Canadian province of Nova Scotia. The Acadian Forest Region is a temperate‐boreal transitional forest zone in eastern Canada and northeastern United States (Rowe, 1972; Taylor et al., 2020; Figure 1). Therein, species that typically grow in a temperate climate zone can be found alongside species that typically grow in a boreal climate zone. The common Acadian species *Acer rubrum* (commonly known as red maple), *Betula papyrifera* (white/paper birch) and *Abies balsamea* (balsam fir) have contrasting geographic distributions in North America. *Acer rubrum* is a more temperate‐climate‐suited species that can be found growing as far south as Florida. *Betula papyrifera* and *Abies balsamea* are more boreal‐climate‐suited species that are relatively uncommon south of the midwestern US (McKenney et al., 2007, 2011, 2014).

The Acadian Forest Region presents an opportunity for monitoring the in‐situ effects of climate change through leaf phenology for both temperate and boreal‐typical species. Boreal species within the Acadian Forest Region such as *Betula papyrifera* and *Abies balsamea* are near the southern limits of their biogeographical range, while temperate species within the Acadian Forest Region such as *Acer rubrum* are near the northern limits of their range (Fisichelli et al., 2014; Pearson & D'Orangeville, 2022). Phenology is a trait that constrains where species can survive, as poorly timed phenology can lead to damage from changing environmental conditions, leading to often greater freeze injury risk for non‐native species (Vitasse et al., 2014; Zanne et al., 2018). Trees which are located near their range limits may be more susceptible to environmental change (Körner et al., 2016; Wang et al., 2021). The Acadian Forest Region is, therefore, especially vulnerable to future changes in temperature and moisture regimes and models have predicted a compositional decline of boreal species due to warming temperatures outside of the optimal biogeographical climate envelopes for these species (Taylor et al., 2017).

In addition, the Acadian Forest Region is subject to extreme weather in the form of hurricanes that lead to windthrow of shallow‐rooted coniferous species such as *Abies balsamea* (Taylor et al., 2019, 2020). If the timing of fall leaf senescence is further delayed in the future, this could also make broadleaf species more susceptible to wind damage due to the added surface area (Gong et al., 2021). In the spring, increased climate variability leads to an increased risk of leaf‐damaging frost events, which is compounded by the already highly dynamic nature of weather patterns in the maritime region of Canada (Augspurger, 2013; Garbary & Hill, 2021; Steenberg et al., 2013). Trees within the Acadian Forest Region may also be at risk of deleterious drought effects as climate models predict an increased frequency and intensity of droughts, and phenology may play an important role in determining drought resilience (Pearson & D'Orangeville, 2022; Sánchez‐Pinillos et al., 2022). The Acadian Forest Region therefore presents a unique and complex forest ecosystem, and better understanding of the leaf phenology of species therein and the potential effects of climate change are needed to predict future ecology and carbon uptake.

Understandings and predictions of future leaf phenology patterns in the eastern Acadian Forest Region are limited due to a lack of observational data, compounded by a highly variable climatic regime (Garbary & Hill, 2021; MacLean et al., 2022; Pearson & D'Orangeville, 2022; Steenberg et al., 2013; Taylor et al., 2020). A study comparing climate normals across Nova Scotia from 1961 to 1990 and 1991 to 2020 found that warming in the autumn has been more pronounced relative to spring, with a larger relative increase in the number of frost‐free days in autumn (Garbary & Hill, 2021). Therefore, leaf senescence observations and modelling are crucial in addition to spring leaf emergence to understand the entire growing season phenology implications of climate change for the Acadian Forest Region. Inter‐continental scale studies have found differing controls of phenology in North America versus Europe and Asia due to historical weather patterns (Zohner et al., 2020). Even within North America, phenological responses to environmental drivers and cues vary regionally (Melaas et al., 2016). This suggests that regional species‐specific observations are needed to develop confident predictions of the response of vegetation to climate change throughout the 21st century for the Acadian Forest Region.

To better understand the environmental controls of leaf phenology for Acadian Forest Region tree species, we used phenocams to monitor the leaf phenology of three tree species across a natural climate gradient in the Canadian province of Nova Scotia throughout the 2019–2022 growing seasons. We also accessed records of leaf phenology across North America using the PhenoCam Network database. We selected the temperate‐climate‐suited species *Acer rubrum* as well as the more boreal‐climate‐suited species *Betula papyrifera* and *Abies balsamea*. These species are common to the Acadian Forest Region and currently monitored throughout the Acadian Phenocam Network and the PhenoCam Network. In this study, we aim to parameterize a variety of species‐specific process‐based models of leaf phenology and simulate leaf phenology and growing season length for *Acer rubrum*, *Betula papyrifera* and *Abies balsamea* under future climate change scenarios.

## 2 MATERIALS AND METHODS

### 2.1 Acadian Phenocam Network

**FIGURE 2:**
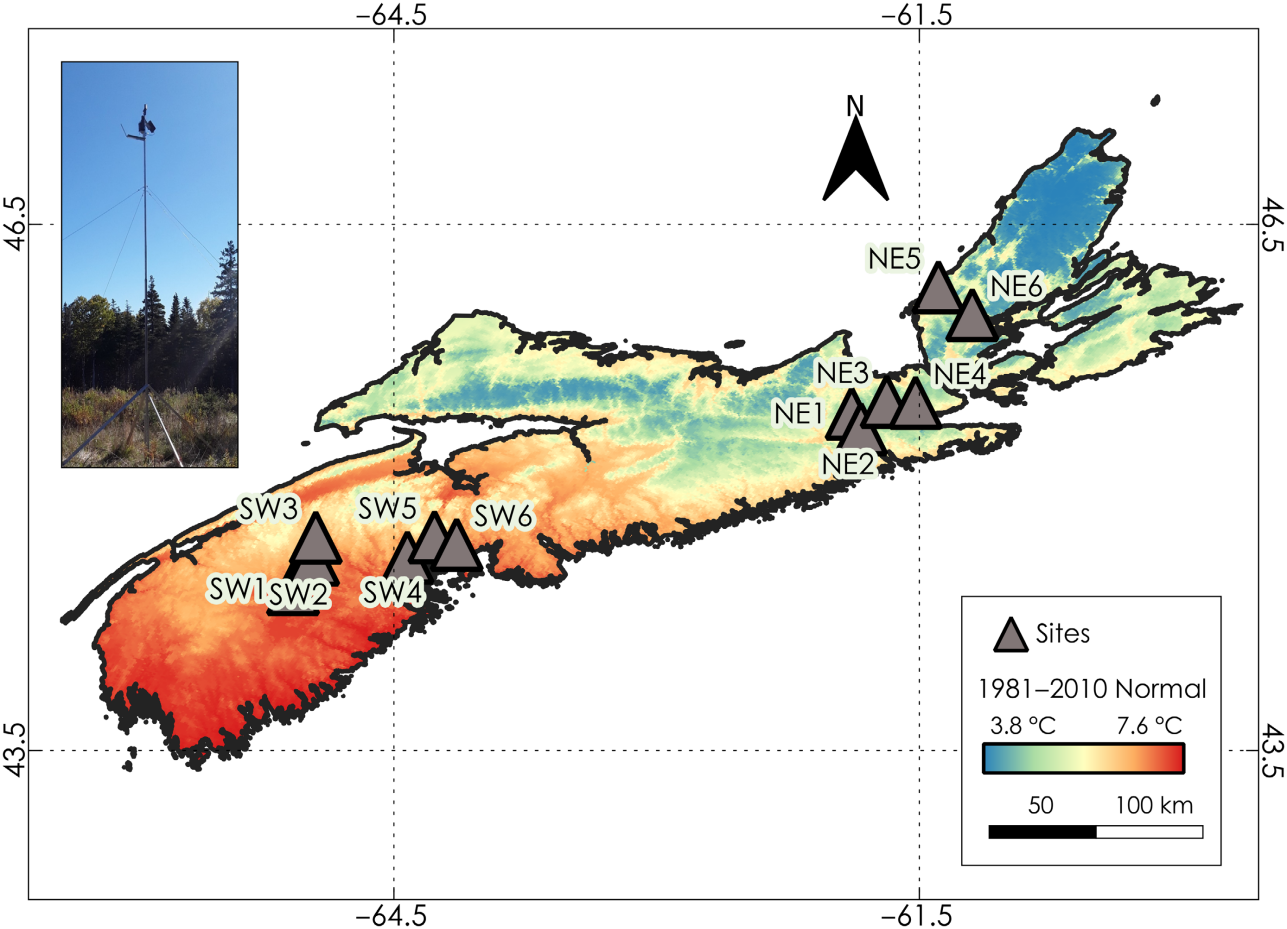
Location of sites within the Acadian Phenocam Network with respect to the 1981–2010 temperature normal. An example station is shown on the top left. The spatial interpolation of mean annual air temperature normals calculated from 1981 to 2010 was obtained from Environment and Climate Change Canada (2022), McKenney et al. (2013) and Price et al. (2011).

To monitor the leaf phenology of Acadian tree species we installed phenocams at 12 sites in the Canadian province of Nova Scotia before the onset of the 2019 growing season (Figure 2). These selected sites were upland, zonal forest sites with sufficient soil nutrients and moisture profiles to support long‐lived, late‐successional species and forests where successional pathways are dictated by climate and not constrained by site conditions (Baldwin et al., 2019). The ecosystem types selected—called ecosites in Nova Scotia's Forest Ecosystem Classification system (Neily et al., 2013)—had both intermediate soil moisture regimes (i.e. fresh) and soil nutrient levels. Mixedwood stands are common on these sites and include broad‐leafed species like *Acer rubrum* (red maple), *Betula alleghaniensis* (yellow birch) and *B. papyrifera* (white/paper birch) and conifer species like *Abies balsamea* (balsam fir) and *Picea rubens* (red spruce). These phenocams were operational throughout the 2019–2020 growing seasons, with several observation gaps in the autumn of 2019 and spring of 2020 due to camera malfunctioning. In the 2020 growing season, we replaced these cameras with cellular trail cameras. Overall, we observed leaf emergence over the 2019–2022 growing seasons and leaf senescence over the 2019–2021 growing seasons. The elevation for our sites ranges from 88 to 322 m above sea level, with most sites located below 200 m.

The technical specifications of each camera type in the Acadian Phenocam Network can be found in Table 1. The 12 cameras installed in 2019 were Moultrie M‐50 trail cameras (https://www.moultriefeeders.com/m‐50‐game‐camera). These cameras were then replaced with Spypoint Link‐Evo (https://www.spypoint.com/en/support/cellular‐trail‐camera/product‐link‐evo.html) cameras. Each camera was solar‐powered, north‐facing and mounted at the top of a 6‐m tower with a horizontal or else tilted ~5° downward landscape view, depending upon the local canopy situation. To ensure stability in the field of view, we used galvanized guy wire cables, aluminum angle tower leg braces and a fixed aluminum angle base. All images were retrieved either remotely using the Python selenium package (Python Software Foundation, 2022; Spypoint cameras) or manually (Moultrie cameras) and catalogued by date and site. Regions of interest (ROIs) were delineated to encompass each distinct individual within the field of view of each camera, as well as the reference panel, using the phenopix package in R version 4.2.1 (Filippa et al., 2016; R Core Team, 2022). The time series of images for each site were reviewed to ensure ROIs were delineated without interference from background elements. The species identification of each ROI was confirmed manually in the field. We classified trees with heights below 5 m as immature and excluded these from analyses, as these tend to exhibit an earlier leaf emergence than mature or canopy‐height conspecific trees, occluding climatic influences (Vitasse & Basler, 2014).

**TABLE 1:** Imagery specifications for cameras utilized across the Acadian Phenocam Network. Image resolutions are shown with pixel and megapixel (MP) dimensions.

Camera	Bits per channel	Resolution	Daily image frequency
Spypoint Link‐Evo	8	1080 × 1920 (2 MP)	1–3
Moultrie M‐50	24	3420 × 6080 (20 MP)	6
Brinno	24	1280 × 720 (0.9 MP)	6

**FIGURE 3:**
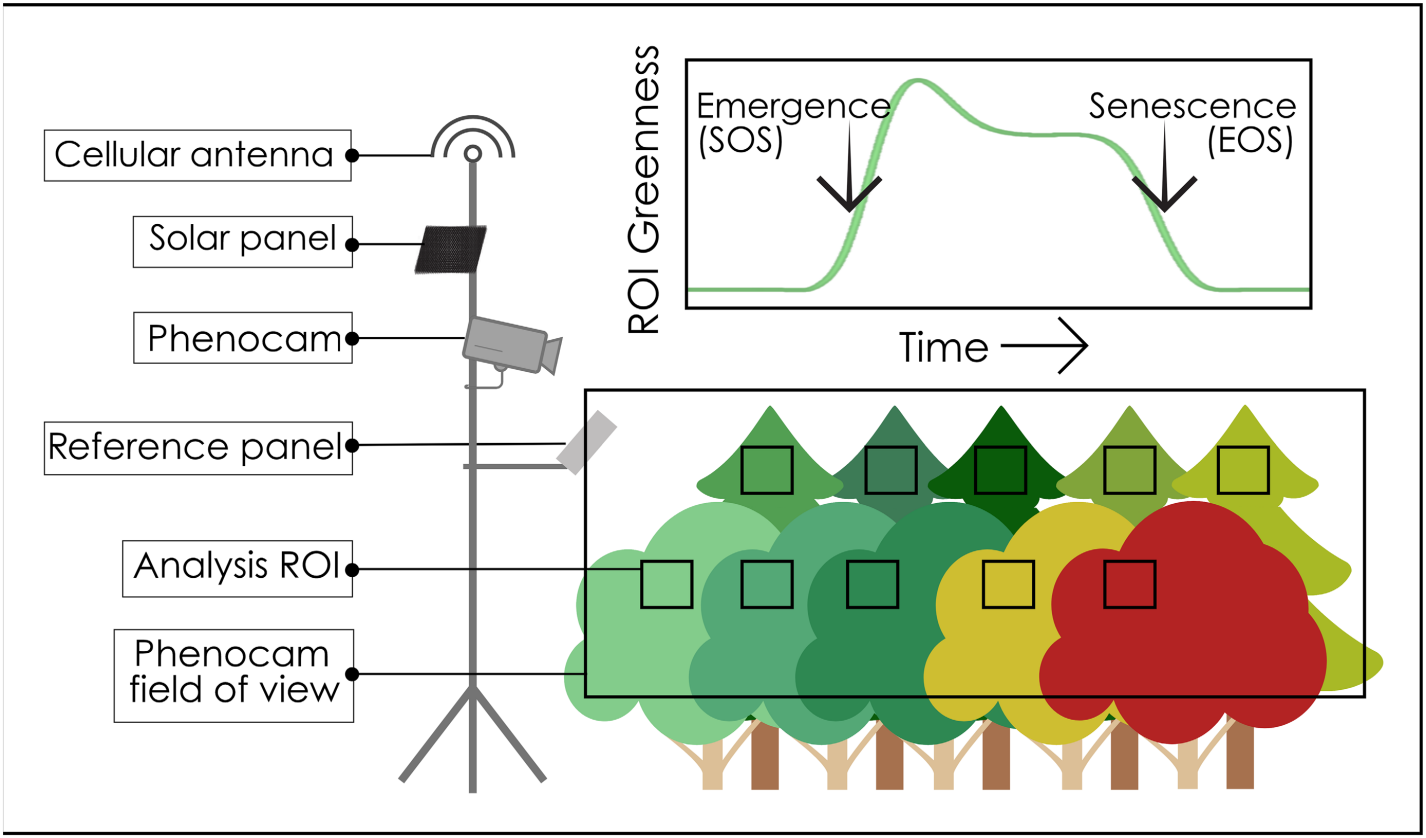
Summary of the station infrastructure (left) and phenology extraction process (right) for Acadian Network phenocams. Here, leaf emergence is synonymous with the start of season (SOS) and leaf senescence with the end of season (EOS).

Slight shifts in the field of view of each camera due to station maintenance over time were accommodated by creating separate analysis ROI coordinates for images before and after each shift using the ‘locator()’ function in the graphics package in R (R Core Team, 2022). Where a tilt in the field of view was detected, new ROIs were carefully delineated to match the targets of the ROIs from the previous field of view. The ‘extractVIs()’ function in the phenopix package was used to extract average red, green and blue colour channel intensity values within each ROI for each image. To extract the greenness time series, we calculated the green chromatic coordinate (*G*_CC_) or relative greenness as is shown in Equation 1.

(1) $$G_{\mathrm{CC}}=\frac{B_{G}}{B_{G}+B_{R}+B_{B}}$$

where *B*_G_ corresponds to the intensity (brightness) of the green colour channel, *B*_R_ to the intensity of the red colour channel, and *B*_B_ to the intensity of the blue colour channel. The *G*_CC_ represents the intensity of the green colour channel versus the total intensity of all colour channels. We then filtered time series by three‐day moving window 50th percentiles of *G*_CC_ values to remove both high and low outliers (Peltoniemi et al., 2018; Richardson, Hufkens, Milliman, Aubrecht, Chen, et al., 2018; Richardson, Hufkens, Milliman, & Frolking, 2018). For further noise reduction, we applied an adapted version of the PhenoCam Network protocols. We exchanged outliers detected as four times greater than the standard deviations of residuals for the upper threshold and two times greater than the standard deviations of residuals for the lower threshold with locally estimated scatterplot smoothing (LOESS) curve‐fitted values to further prioritize the removal of anomalous *G*_CC_ declines (Richardson, Hufkens, Milliman, Aubrecht, Chen, et al., 2018; Seyednasrollah, Young, Hufkens, Milliman, Friedl, Frolking, & Richardson, 2019). For smoothing over the dormant period, we exchanged original dormant period G_CC_ values with that of the dormant season mode, calculated as the lowest local maxima in a density plot of *G*_CC_ values for a given year. We calculated the timing of leaf emergence and senescence as 50% of the amplitude of rising and falling *G*_CC_ curves (Figure 3). Growing season length was calculated as the time between leaf emergence and senescence. To obtain phenology estimates at the site level, we selected sites with at least three individuals of a given species present and averaged each individual phenocam‐derived phenology date to produce a site‐level observation for each species and site‐year, which are shown in Section A2: Tables A5 and A6 in Appendix A for leaf emergence and senescence, respectively.

The Moultrie M‐50 and Spypoint Link‐Evo trail camera models deployed in this study do not have the option to fix the image white balance. Spurious vegetation transition signals may arise due to an automatic white balance (Richardson, Hufkens, Milliman, Aubrecht, Chen, et al., 2018; Seyednasrollah, Young, Hufkens, Milliman, Friedl, Frolking, & Richardson, 2019). To ensure that the colour channel patterns observed through the cameras across our sites were due to changes in leaf canopy development rather than colour scaling artefacts, we utilized several means of validation and of quality control: (1) installation of grey non‐reflective reference panels in the field of view of all cameras in 2020 onwards and normalization of vegetation *G*_CC_ time series following Delpierre et al. (2020) and Jacobs et al. (2009), (2) filtering threshold amplitude of *G*_CC_ time series twice that of the reference panel, (3) comparison of curve‐estimated phenology to phenology obtained from daily (May) to weekly (June onwards) manual ground observations for three *Populus tremuloides* individuals at an external site in 2021, (4) comparison of curve‐estimated phenology for 15 individuals to the estimation of leaf phenology via visual inspection of images for 2019, 2020 and 2021 site‐years (Ahrends et al., 2009; Klosterman et al., 2014; Kosmala et al., 2016; Peltoniemi et al., 2018), and (5) comparison of leaf phenology derived from a Spypoint camera to that of a fixed white balance Brinno camera (https://brinno.com/pages/product‐tlc200pro) at an external site. For both manual field and visual observations, we considered leaf emergence to occur when most leaves had emerged entirely from bud scales such that leaf midribs were visible and leaf senescence to occur when most leaves had begun to show autumn colouration. Curve‐estimated leaf emergence dates for the three *Populus tremuloides* individuals at the external site for the Spypoint Link‐Evo camera were equal to the reference manually observed date (May 22nd, 2021, for all individuals) and the Brinno camera estimate was 4 days early. Curve‐estimated leaf emergence values had a correlation of 0.78 with the 15 visual estimates, while curve‐estimated leaf senescence values had a correlation of 0.74. This performance was in line with previous studies which have shown correlation values of 0.52–0.99 for leaf emergence 0.61–0.99 for leaf senescence (Ahrends et al., 2008, 2009; Berra et al., 2016; Browning et al., 2017; Delpierre et al., 2020; Keenan et al., 2014; Klosterman et al., 2014; Klosterman & Richardson, 2017; Kosmala et al., 2016; Peltoniemi et al., 2018; Richardson, Hufkens, Milliman, Aubrecht, Chen, et al., 2018; Seyednasrollah et al., 2021; Seyednasrollah, Young, Hufkens, Milliman, Friedl, Frolking, & Richardson, 2019; Wingate et al., 2015; Xie, Civco, & Silander Jr, 2018; Zhang et al., 2020).

### 2.2 PhenoCam Network

To ensure our phenology model training dataset was representative of future climate space, we employed phenocam ROI site‐year observations from the North American PhenoCam Network. For information on PhenoCam Network protocols refer to Seyednasrollah, Young, Hufkens, Milliman, Friedl, Frolking, and Richardson (2019) and Richardson, Hufkens, Milliman, Aubrecht, Chen, et al. (2018). Regions of interest from PhenoCam cameras are delineated to characterize the dominant vegetation in each field of view, and in some cases, several ROIs are defined to distinguish between different plant functional types such as evergreen needleleaf versus deciduous needleleaf (Richardson, Hufkens, Milliman, Aubrecht, Furze, et al., 2018). The *G*_CC_ is then calculated as shown in Equation 1 from red, green and blue colour channel intensity values within each ROI for each image to produce greenness time series. For compatibility with Acadian Phenocam Network observations, we extracted the timing of 50% amplitude in the rising and falling portion of 3‐day 50th percentile filtered *G*_CC_ records from the PhenoCam V2.0 dataset, which includes observations up until the end of the 2018 growing season (Seyednasrollah, Young, Hufkens, Milliman, Friedl, Frolking, Richardson, Abraha, et al., 2019). To populate our training dataset for each tree species, we selected phenocam records from the PhenoCamV2.0 dataset which had one of our species as the dominant species in the field of view (Table 2).

**TABLE 2:** Summary of observations from the Acadian Network and PhenoCam Network. Each observation corresponds to one phenocam site‐year.

Species	Leaf Phenophase	Total number of site‐years	Total Acadian site‐years	Total PhenoCam site‐years
*Acer rubrum*	Emergence	278	34	244
*Betula papyrifera*	Emergence	89	20	69
*Abies balsamea*	Emergence	43	28	15
*Acer rubrum*	Senescence	237	22	215
*Betula papyrifera*	Senescence	82	14	68
*Abies balsamea*	Senescence	29	15	14

### 2.3 Leaf emergence models

We explored a variety of leaf emergence models with varying degrees of complexity in terms of environmental drivers, including thermal forcing, chilling exposure and photoperiod from Hufkens et al. (2018) and Basler (2016). Each model generally simulates an accumulation until a critical threshold is reached and leaf emergence occurs, with a parameterized starting date for the accumulation of drivers. Four of the models simulate a release from ecodormancy only, using either temperature forcing alone or in combination with photoperiod as drivers. The Thermal Time model (TT) accumulates forcing above a base temperature in a linear fashion until a critical threshold is reached and leaf emergence occurs (Basler, 2016; De Réaumur, 1735; Hufkens et al., 2018; Wang, 1960). The Thermal Time with Sigmoidal Temperature Response model (TTs) also accumulates forcing above a base temperature until a critical threshold is reached and leaf emergence occurs, though with a sigmoidal accumulation function (Basler, 2016; Hänninen, 1990; Hufkens et al., 2018; Kramer, 1994). The Photo‐Thermal Time model (PTT) accumulates forcing above a base temperature in a linear fashion adjusted by daylength (Basler, 2016; Črepinšek et al., 2006; Hufkens et al., 2018; Masle, 1989). The Photo‐Thermal Time with Sigmoidal Temperature Response model (PTTs) also accumulates forcing above a base temperature with a sigmoidal function adjusted by daylength until a critical threshold is reached and leaf emergence occurs (Basler, 2016; Črepinšek et al., 2006; Hänninen, 1990; Hufkens et al., 2018; Kramer, 1994; Masle, 1989). The M1 model is similar to the Photo‐Thermal Time model, though with an additional exponential constant (Basler, 2016; Blümel & Chmielewski, 2012; Hufkens et al., 2018).

Two models simulate a release from endodormancy and ecodormancy with a combination of temperature forcing and chilling as drivers. The Alternating Model (AT) accumulates forcing and chilling exposure without the stipulation that chilling requirements are to be met prior to the onset of forcing accumulation. Within the Alternating Model each day can contribute to either requirement until accumulated forcing has surpassed a critical threshold which is altered by chilling exposure, and leaf emergence occurs (Basler, 2016; Cannell & Smith, 1983; Hufkens et al., 2018; Murray et al., 1989). The Sequential Model (SQ) assumes that chilling requirements are fulfilled prior to the onset of forcing accumulation with a bell‐shaped chilling temperate response function. Once a critical threshold in chilling accumulation is reached, forcing accumulates until another critical threshold is reached and leaf emergence occurs (Basler, 2016; Hänninen, 1990; Hufkens et al., 2018; Kramer, 1994). Finally, the Dormphot model (DP) simulates dormancy induction, endodormancy release and ecodormancy release with a combination of drivers including chilling, forcing and daylength. Within the Dormphot model, dormancy induction occurs once accumulated cool temperatures and shortening daylengths in the fall reach a combined critical threshold. Once dormancy has been induced, chilling accumulates and adjusts a parameter that governs daylength and forcing accumulation until a critical threshold is reached and leaf emergence occurs (Basler, 2016; Caffarra et al., 2011; Hufkens et al., 2018).

All leaf emergence models were applied using the phenor package in R with species‐specific training and validation for each available site‐year (Hufkens et al., 2018). Model parameterization for each species was optimized using the initial parameter ranges from Hufkens et al. (2018) and general simulated annealing with the optimize_parameters function within the phenor package which is an extension of the GenSA optimization function within the GenSA package in R (Xiang et al., 2013). General simulated annealing is a technique of optimization that is analogous to the process of metal cooling (Chuine et al., 2000). The GenSA function is based on the Boltzmann machine and Cauchy machine simulated annealing approaches (Hufkens et al., 2018; Tsallis & Stariolo, 1996). General simulated annealing was constrained with a maximum of 50,000 iterations and a starting temperature of 10,000 for each annealing to achieve a global minimum in root mean squared error (RMSE). Equations and optimal parameters for each model from this process are shown in Section A1: Tables A1 and A2 in Appendix A, respectively.

### 2.4 Leaf senescence models

We explored a variety of leaf senescence models with various configurations of environmental drivers including temperatures, photoperiod and the preceding estimated leaf emergence date from Liu et al. (2020). As with the leaf emergence models, each leaf senescence model generally simulates an accumulation until a critical threshold is reached and leaf senescence occurs, though with a parameterized or fixed July 1st starting date. The White model (WM) is an exception that does not involve accumulation and instead uses instant senescence triggers based on cooling temperatures and shortening daylength or else extreme cold temperatures (Liu et al., 2020; White et al., 1997). The Delpierre model (DM) accumulates cool temperatures and shortening daylength at a rate controlled by driver‐specific weighting parameters until a critical threshold is reached and lead senescence occurs (Delpierre et al., 2009; Liu et al., 2020). The Jeong Model (JM) accumulates cool temperatures once daylength is sufficiently short until a critical threshold is reached and leaf senescence occurs (Jeong & Medvigy, 2014; Liu et al., 2020). The Dormphot Dormancy Induction Model (DPDI) is identical to the dormancy induction simulation within the full Dormphot leaf emergence model used above. Leaf senescence or dormancy therein occurs once cooling temperatures and shortening daylength accumulate to a critical value (Caffarra et al., 2011; Hufkens et al., 2018; Liu et al., 2020). Two models, the Delpierre model with Preceding Spring Leaf Emergence (DMs) and the Dormphot Dormancy Induction Model with Preceding Spring Leaf Emergence (DPDIs), include the influence of the preceding leaf emergence timing which has been found to exert a constraint of leaf senescence timing. For these models, the Photo‐Thermal Time with Sigmoidal Temperature Response model is used to provide an estimated leaf emergence timing that incorporates both forcing and daylength (Keenan & Richardson, 2015; Liu et al., 2020). The timing of the preceding leaf emergence relative to the long‐term average estimated from a 30‐year daily average temperature window influences the critical threshold for leaf senescence for these two models.

The parameter ranges for each leaf senescence model were determined by reviewing local climate data, the available literature for each model and the range of optimal values from Liu et al. (2020). All leaf senescence models were applied in R with species‐specific training and validation for each available site‐year. As with the leaf emergence models, senescence model parameterizations were optimized for each species using general simulated annealing with a maximum of 50,000 iterations and a starting temperature of 10,000 for each annealing to find model parameters that corresponded to a global minimum RMSE. Equations and optimal parameters for each model from this process are shown in Section A1: Tables A3 and A4, respectively in Appendix A.

### 2.5 Model performance evaluation

To evaluate the performance of each model for each species, we calculated RMSE between the model predicted and that of phenocam‐derived leaf emergence and senescence timings for each site‐year observation with a pooled‐sample or global validation. We calculated the Null model RMSE as the RMSE between observed values and the average observed training dataset value to compare model performances to the assumption of a fixed mean value. We considered the model with the lowest RMSE to be the best‐performing model for each validation exercise.

To examine the regional transferability of each model, we performed a smaller scale calibration and validation with observations just from the Acadian Phenocam Network. To examine the regional specificity of the models, we also trained the models with all observations and then validated the models with just observations from the Acadian Phenocam Network. We also calculated the bias between model‐estimated and observed phenophases for each validation exercise to examine potential systematic over‐ and under‐estimations. To examine globally parameterized model performances for warm site‐years and for cold‐site years, we selected validation site‐years with annual average temperatures within the top 25th percentile of annual average temperatures for warm site‐years and within the lower 25th percentile for cold site‐years. We then computed the RMSE and bias for these site‐years for globally parameterized models. To examine how well these models performed with independent validation, we also performed a leave‐one‐out cross‐validation and a k‐fold cross‐validation. For efficiency with these two validations, each model was parameterized with a maximum of 4000 iterations. The value of ‘k’ was allowed to vary such that each sample group had five or more samples, which is an effective model quality assessment approach with a range in dataset sizes (Jiang & Wang, 2017; Yadav & Shukla, 2016).

### 2.6 Obtaining driver data

To train each phenology model, we obtained daily weather data for each site‐year in both the Acadian and PhenoCam Network datasets up until 2021 from the Daymet: Daily Surface Weather Data on a 1‐km Grid for North America, Version 4 R1 dataset using the daymetr package in R (Hufkens et al., 2018; Thornton et al., 2022). Long‐term mean temperatures from 1980 to 2021 were also obtained for each site from this dataset using the daymetr package. For January–July of 2022 for the Acadian Network, we obtained daily weather data from the Daymet Version 4 dataset (Thornton et al., 2021; https://daac.ornl.gov/cgi‐bin/dsviewer.pl?ds_id=1904). We used 1 km × 1 km grids with the nearest central coordinates to each site location for each site‐year.

To project leaf phenology for each of our three species for the Acadian Forest Region sites, we simulated leaf phenology from 2001 to 2100 with each leaf species‐calibrated phenology model under the effects of climate warming with three representative concentration pathways (RCPs): RCP 2.6 (low emissions), RCP 4.5 (intermediate emissions) and RCP 8.5 (high emissions). We obtained simulated daily temperature data with the Coupled Model Intercomparison Project 5 (CMIP5) model ensemble of 24 climate models from ClimateData.ca (2022; https://climatedata.ca/download/). Each climate model output was downscaled and bias‐adjusted using the Bias Correction/Constructed Analogues with Quantile delta mapping reordering (BCCAQv2) method (Cannon et al., 2015). We extracted data from the 300‐arc second spatial resolution (1/12°, ~10 km) grid cells with central coordinates closest to our site locations.

## 3 RESULTS

### 3.1 Leaf phenology training data

**FIGURE 4:**
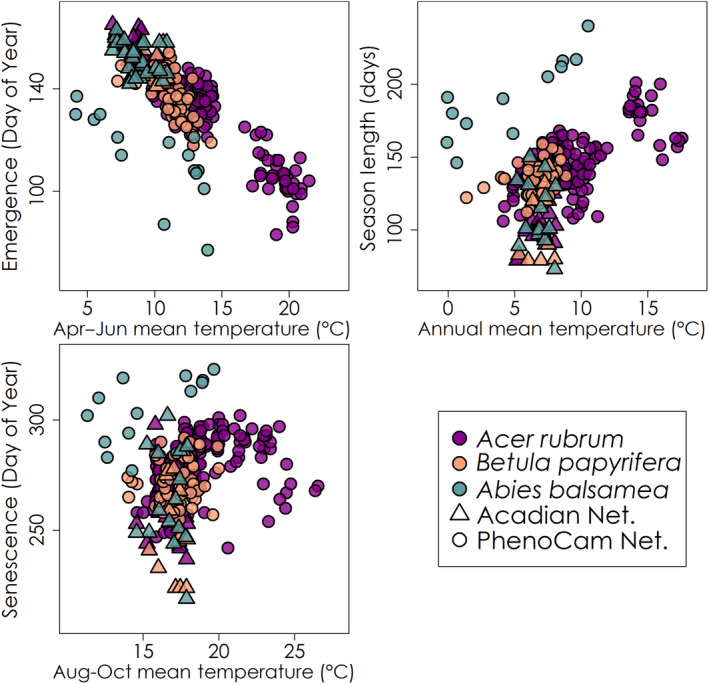
Leaf phenology patterns according to seasonal or annual temperature averages for both the Acadian and PhenoCam Networks.

Leaf phenology patterns in relation to temperature were similar for the Acadian and PhenoCam Networks (Figure 4). Within the Acadian Phenocam Network, each species generally showed a spatial pattern in leaf emergence dates reflecting the climate gradient used in their establishment, with later emergence at the colder northeastern sites and earlier at the warmer southwestern sites. In contrast, leaf senescence dates for each species did not exhibit a distinct climate pattern. Surprisingly, several sites in the warmer region of the Acadian Network had earlier leaf senescence dates than that of the colder region, suggesting the timing of preceding leaf emergence may have had an important influence on the timing of leaf senescence. Leaf emergence was earlier for warmer springs for all species, with a significant linear relationship (adjusted *R* ^2^: .81, *p* < .001 for *Acer rubrum*, adjusted *R* ^2^: .56, *p* < .001 for *Betula papyrifera*, adjusted *R* ^2^: .25, *p* < .001 for *Abies balsamea*). The more boreal‐typical species *Betula papyrifera* and *Abies balsamea* had reduced coverage of the growing season temperature ranges relative to *Acer rubrum*, though under similar temperatures tended to have similar or earlier timings of leaf emergence and senescence. Under similar temperatures, *Abies balsamea* in the Acadian Network tended to have a later leaf emergence in comparison to the PhenoCam Network. In contrast, leaf senescence had no clear linear relationship with late summer‐early autumn temperatures. While there is some overlap in leaf senescence timings for *Abies balsamea* in both networks, within the Acadian Network under similar conditions it tended to be earlier, prompting a reduced season length. Season lengths tended to increase with increasing mean annual temperatures across site‐years, though with values ranging on the order of weeks at a given annual average temperature within and among species. Overall, the Acadian Phenocam Network is situated with cold‐intermediate seasonal temperatures relative to the PhenoCam Network, though these species tend towards later leaf emergence and earlier leaf senescence, leading to a reduced season length.

### 3.2 Leaf emergence models

**FIGURE 5:**
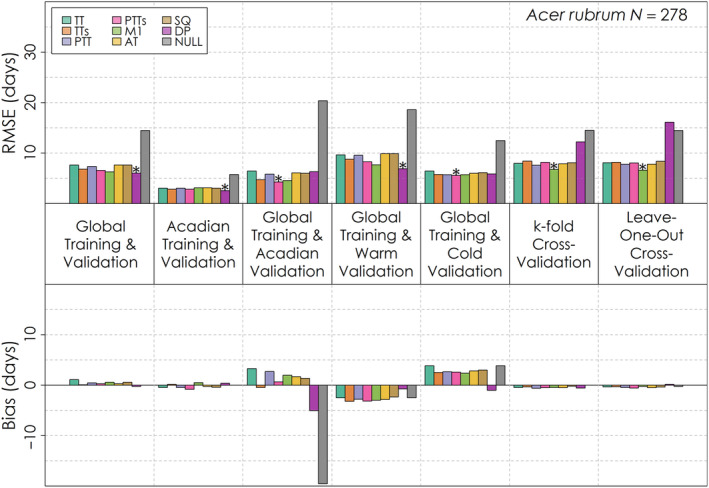
Root mean squared error and mean bias for each of eight leaf emergence models and a Null model for seven different validation exercises for *Acer rubrum*. The total number of *Acer rubrum* leaf emergence observations for is shown on the top right. The model with the lowest root mean squared error for each validation exercise is denoted with an asterisk.

**FIGURE 6:**
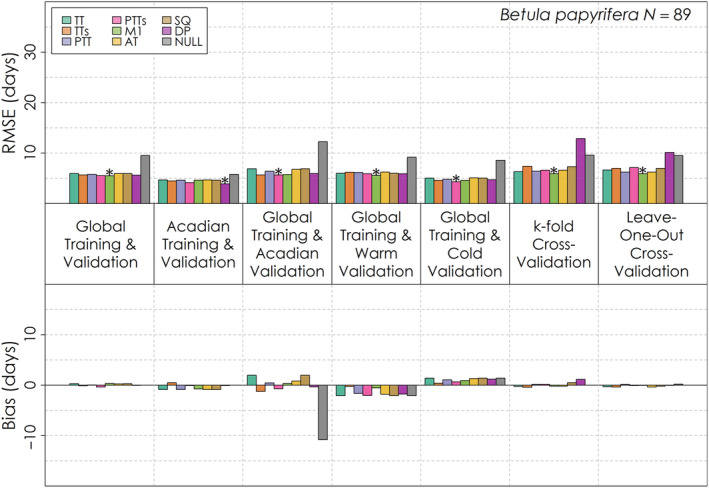
Root mean squared error and mean bias for each of eight leaf emergence models and a Null model for seven different validation exercises for *Betula papyrifera*. The total number of *Betula papyrifera* leaf emergence observations for is shown on the top right. The model with the lowest root mean squared error for each validation exercise is denoted with an asterisk.

**FIGURE 7:**
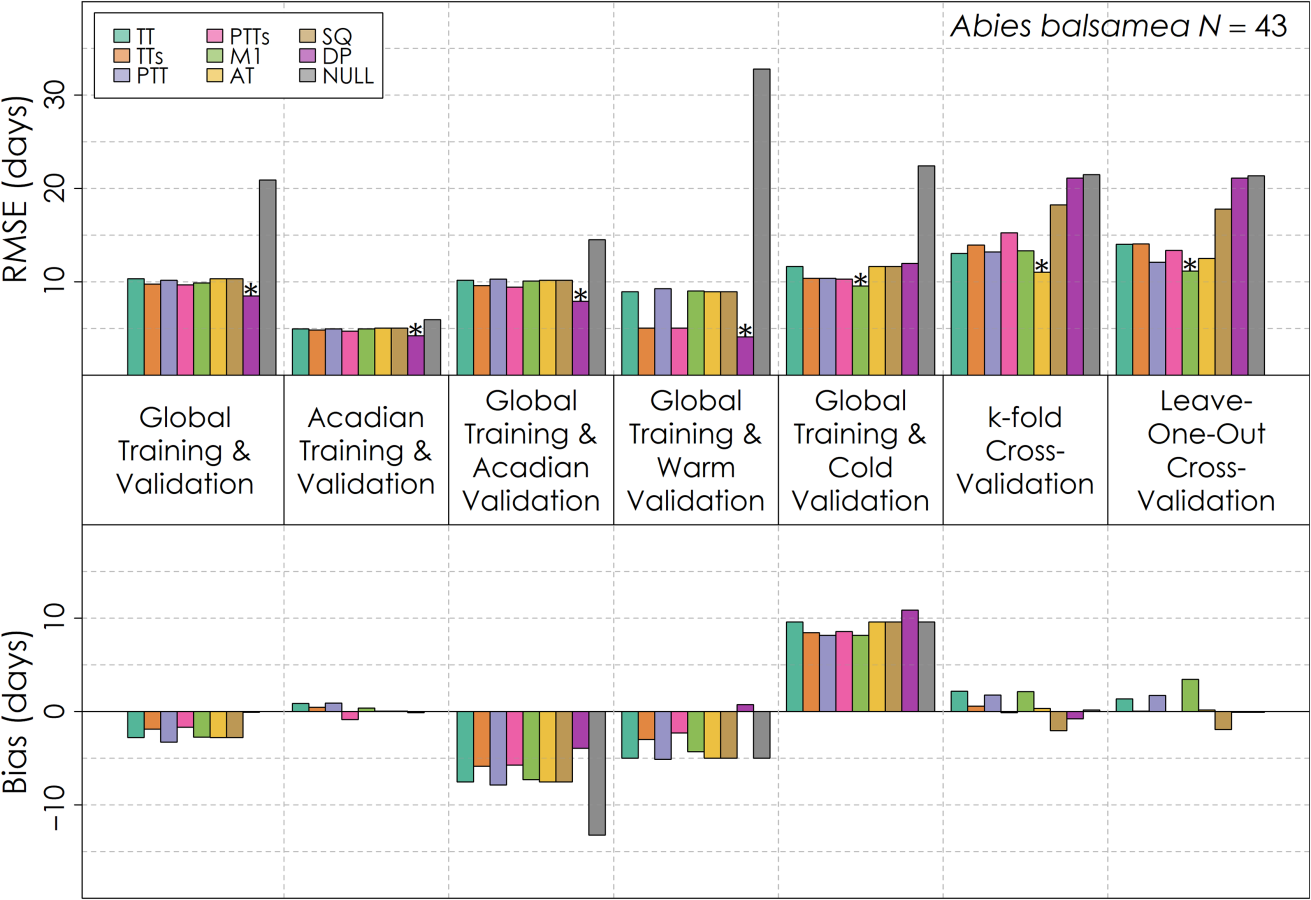
Root mean squared error and mean bias for each of eight leaf emergence models and a Null model for seven different validation exercises for *Abies balsamea*. The total number of *Abies balsamea* leaf emergence observations for is shown on the top right. The model with the lowest root mean squared error for each validation exercise is denoted with an asterisk.

No one leaf emergence model had exceptional performance relative to the other models across species and validation exercises, though all generally managed to outperform the Null model (Figures 5, 6, 7). For the global validation, the Dormphot and M1 models were among the top two models with the lowest RMSE relative to other models for each species. All eight models outperformed the Null model for each species and validation, each process model having typically a week or less in RMSE for *Acer rubrum* (Figure 5) and *Betula papyrifera* (Figure 6) and less than 2 weeks for *Abies balsamea* (Figure 7). Training and validating with just the Acadian Phenocam Network generally prompted a similar pattern in model performance to the global evaluation, though the Dormphot model outperformed the M1 model for *Betula papyrifera*. In contrast, global training and validation with just the Acadian Phenocam Network prompted a different pattern in model performance, favoring the Photo‐Thermal Time with Sigmoidal Temperature Response model for *Betula papyrifera* and *Acer rubrum*, though the Dormphot model for *Abies balsamea*. When predicting leaf emergence for warm site‐years the M1 model was optimal for *Betula papyrifera* while the Dormphot model was optimal for the other species. During cold site‐years, the M1 model was optimal for *Abies balsamea* while the Photo‐Thermal Time with Sigmoidal Temperature Response model was optimal for the other species. For k‐fold and leave‐one‐out cross‐validation, the M1 model was generally optimal, though the Alternating Time model was optimal for k‐fold cross‐validation with *Abies balsamea*. For both *Acer rubrum* and *Betula papyrifera*, the Dormphot model failed to outperform the Null model with one or both of the k‐fold and leave‐one‐out cross‐validations, suggesting the performance of this complex model is sensitive to validation sample size. Overall, optimal models across validation exercises and species were the Dormphot, M1 and Photo‐Thermal Time with Sigmoidal Temperature Response models, suggesting these models are well suited to application across different species when conducting species‐specific parameterizations. For *Acer rubrum* and *Abies balsamea* performance across models ranged by about 2 days, while for *Betula payrifera* RMSE values ranged by less than half a day. Bias varied in direction and magnitude across validations. For *Abies balsamea* (Figure 7), global training and Acadian validation prompted an early bias while for *Acer rubrum* (Figure 5) and *Betula papyrifera* (Figure 6), the same validation prompted a mixed bias. All models had an early bias of prediction for warm site‐years and a late bias for cold site‐years. The simple thermal time model was among the top three models with the greatest absolute bias for a variety of validation exercises across species, including global training and validation, validation with just the Acadian Network, with warm years and with cold years.

### 3.3 Leaf senescence models

**FIGURE 8:**
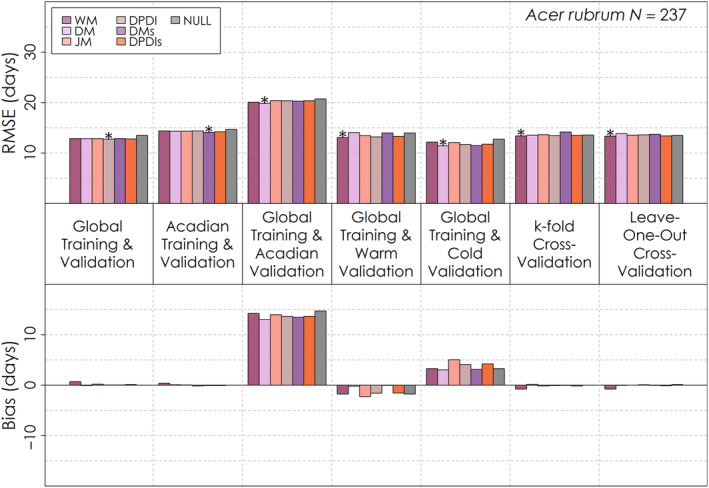
Root mean squared error and mean bias for each of six leaf senescence models and a Null model for seven different validation exercises for *Acer rubrum*. The total number of *Acer rubrum* leaf senescence observations for is shown on the top right. The model with the lowest root mean squared error for each validation exercise is denoted with an asterisk.

**FIGURE 9:**
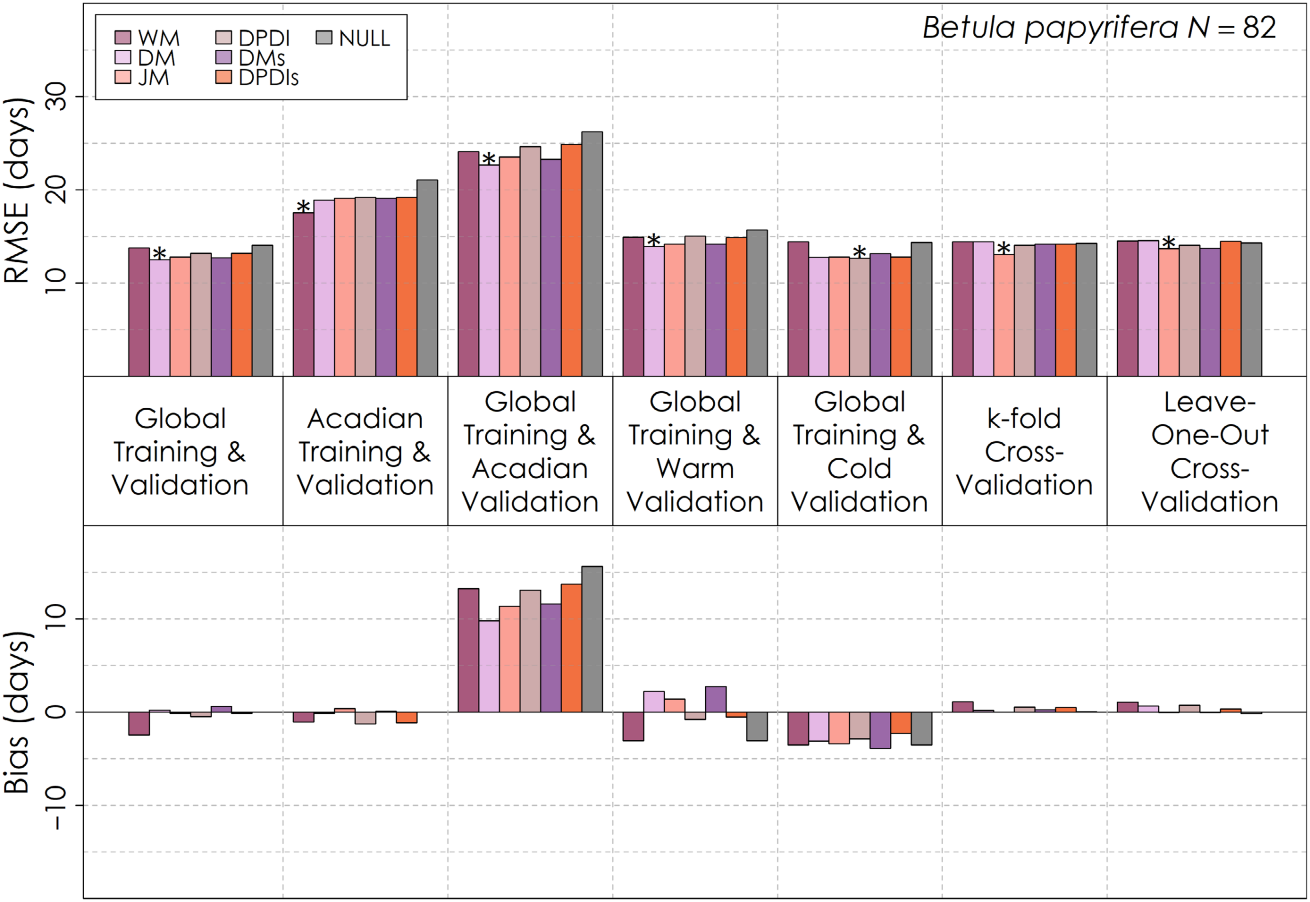
Root mean squared error and mean bias for each of six leaf senescence models and a Null model for seven different validation exercises for *Betula papyrifera*. The total number of *Betula papyrifera* leaf senescence observations for is shown on the top right. The model with the lowest root mean squared error for each validation exercise is denoted with an asterisk.

**FIGURE 10:**
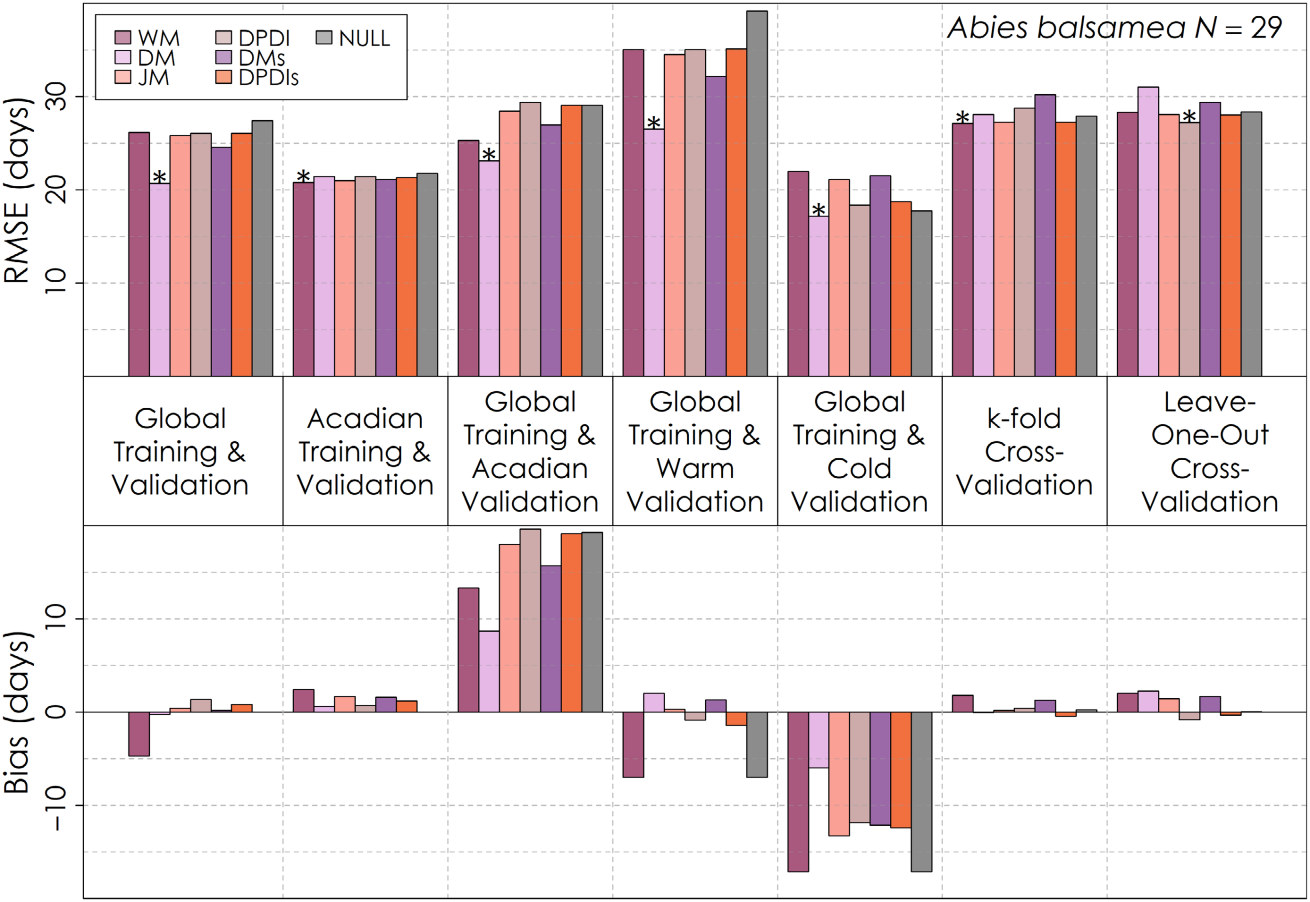
Root mean squared error and mean bias for each of six leaf senescence models and a Null model for seven different validation exercises for *Abies balsamea*. The total number of *Abies balsamea* leaf senescence observations for is shown on the top right. The model with the lowest root mean squared error for each validation exercise is denoted with an asterisk.

Similarly, no one leaf senescence model had exceptional global performance relative to the others, with some failing to outperform the Null model for several validations (Figures 8, 9, 10). For the global validation, the Delpierre model was optimal for *Abies balsamea* (Figure 10) and *Betula papyrifera* (Figure 9), while the Dormphot model with just Dormancy Induction was optimal for *Acer rubrum* (Figure 8). Across models, species and validations, RMSE ranged from less than 2 weeks to about 4 weeks. Model RMSE values were high for each species relative to leaf emergence models and among species highest for *Abies balsamea*. For *Acer rubrum* performance across process models was very similar, ranging by less than half a day, while for *Betula papyrifera* RMSE values ranged by about 1 day, and for *Abies balsamea* ranged by about 5 days. Training and validating with just the Acadian Network favoured the Delpierre with Preceding Spring Leaf Emergence for *Acer rubrum*, and the White model for the other species. When globally trained and validated with just the Acadian Network, the Delpierre model was optimal for all species. For warm site‐years, the White model was optimal for *Acer rubrum* while the Delpierre model was optimal for other species. Alternatively, during cold site‐years, the Delpierre model was optimal for *Acer rubrum* and *Abies balsamea*, while the Dormphot model with just Dormancy Induction was optimal for *Betula papyrifera*. For the k‐fold cross‐validation, the White model was best for *Acer rubrum* and *Abies balsamea*, while the Jeong model was optimal for *Betula papyrifera*. Overall, the Delpierre, White and Dormphot with just Dormancy Induction models were among the top‐performing models for each validation exercise across species. Bias varied in magnitude and direction across validation exercises, and even across species for the same validation exercises in some cases. With global training and validation with just the Acadian Network, each model predicted a late timing of leaf senescence by about one to more than 2 weeks across species, though the Delpierre model had the lowest absolute bias. Models predicted early and late leaf senescence for warm and cold site‐years, respectively, for *Acer rubrum* (Figure 8), though mixed and early leaf senescence for *Abies balsamea* (Figure 10) and *Betula papyrifera* (Figure 9). Despite its occasionally preferable RMSE scores, the simple trigger‐based White model was often among the top three models with the greatest absolute bias across validation exercises for each species.

### 3.4 Projected climate

**FIGURE 11:**
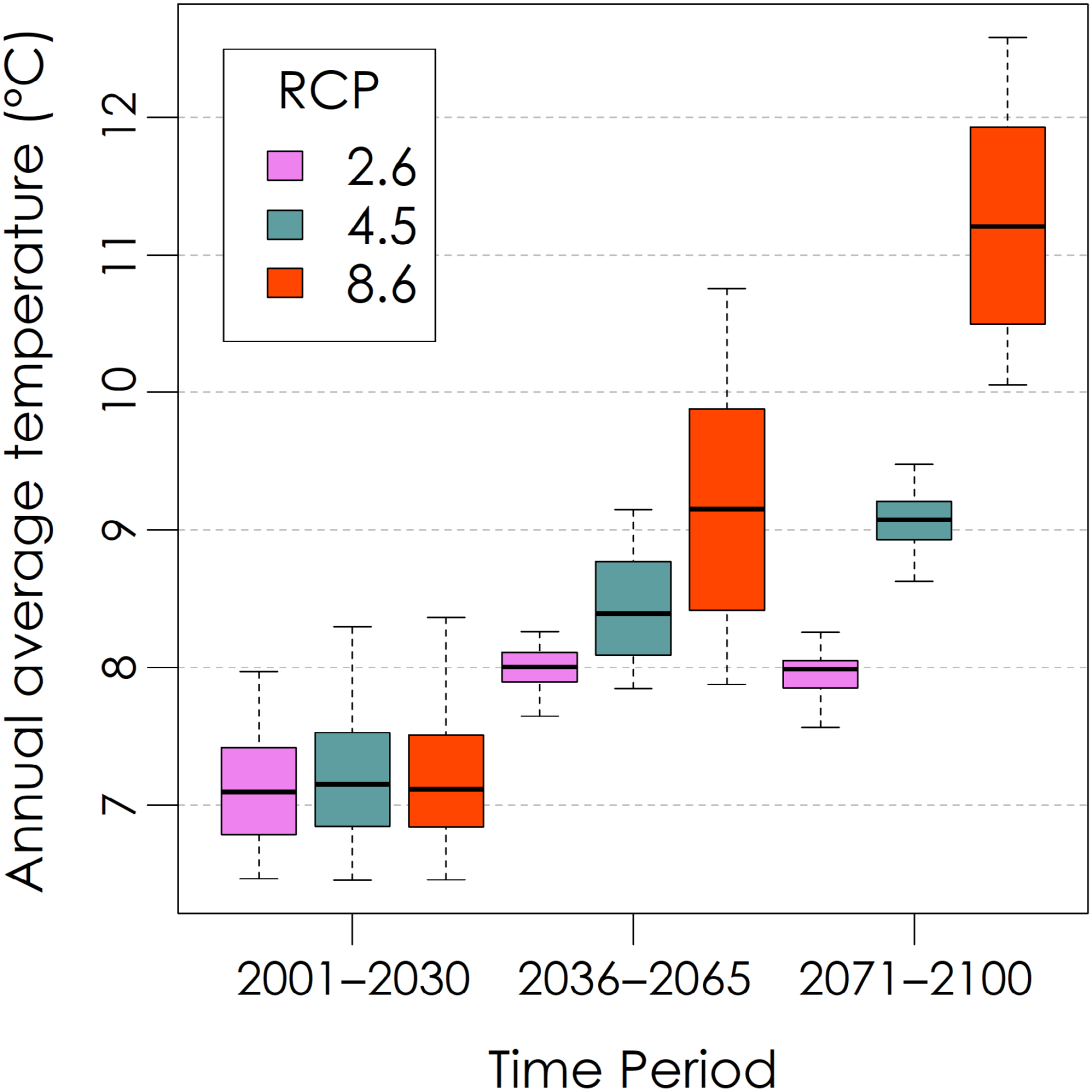
Projected annual average temperatures across sites in the Acadian Phenocam Network.

Under the RCP 8.5 scenario, annual average temperatures in the year 2100 were projected to increase by about 4°C from 7 to 11°C relative to 1990–2020 across our 12 sites (Figure 11). For the RCP 2.6 scenario, a more moderate temperature increase of about 1°C was projected. The projected temperature change for northeastern sites was equivalent to warming them to the temperature of the southwestern sites for each emissions scenario. The projected annual average temperatures for our sites in 2100 under the RCP 8.5 scenario are within the range of training dataset annual average temperatures for *Acer rubrum* (annual average ~4–18°C) and *Abies balsamea* (annual average ~0–11°C), though slightly beyond the range for *Betula papyrifera* (annual average ~1–9°C; Figure 4).

### 3.5 Projected leaf phenology

**FIGURE 12:**
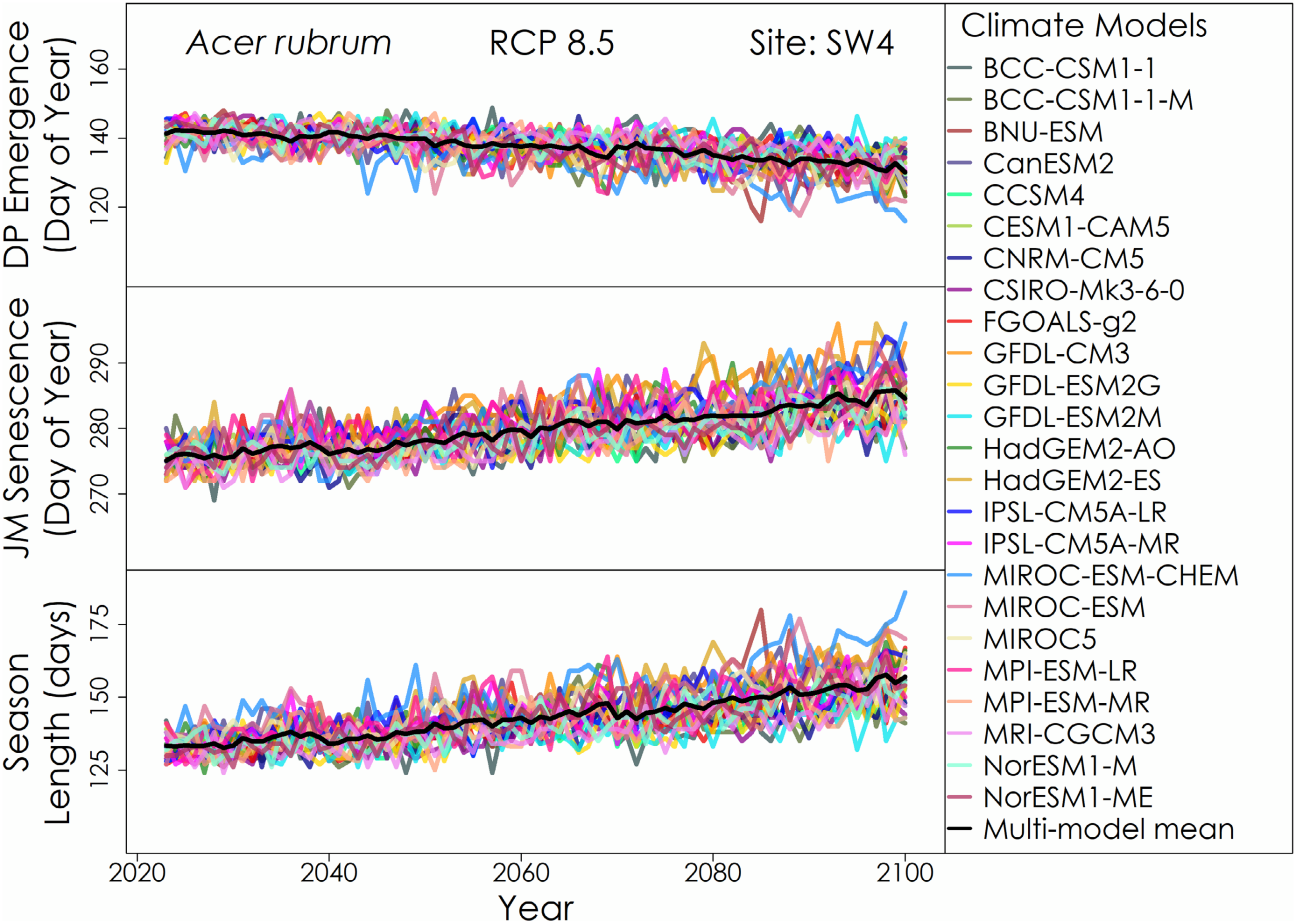
Example time series of predicted *Acer rubrum* leaf emergence and senescence timings and the corresponding length of season with the Dormphot leaf emergence model and the Jeong leaf senescence model for each CMIP5 climate model under RCP 8.5 at site SW4.

For each site, phenology model, species, the projected phenology and season length varied each year throughout the 21st century among climate models (Figure 12). For each phenology model, variation in predicted leaf emergence dates among climate models was generally greater each year than variation in predicted leaf senescence dates. The predicted leaf emergence for some years was anomalous with respect to adjacent years and the longer record for several climate models.

**FIGURE 13:**
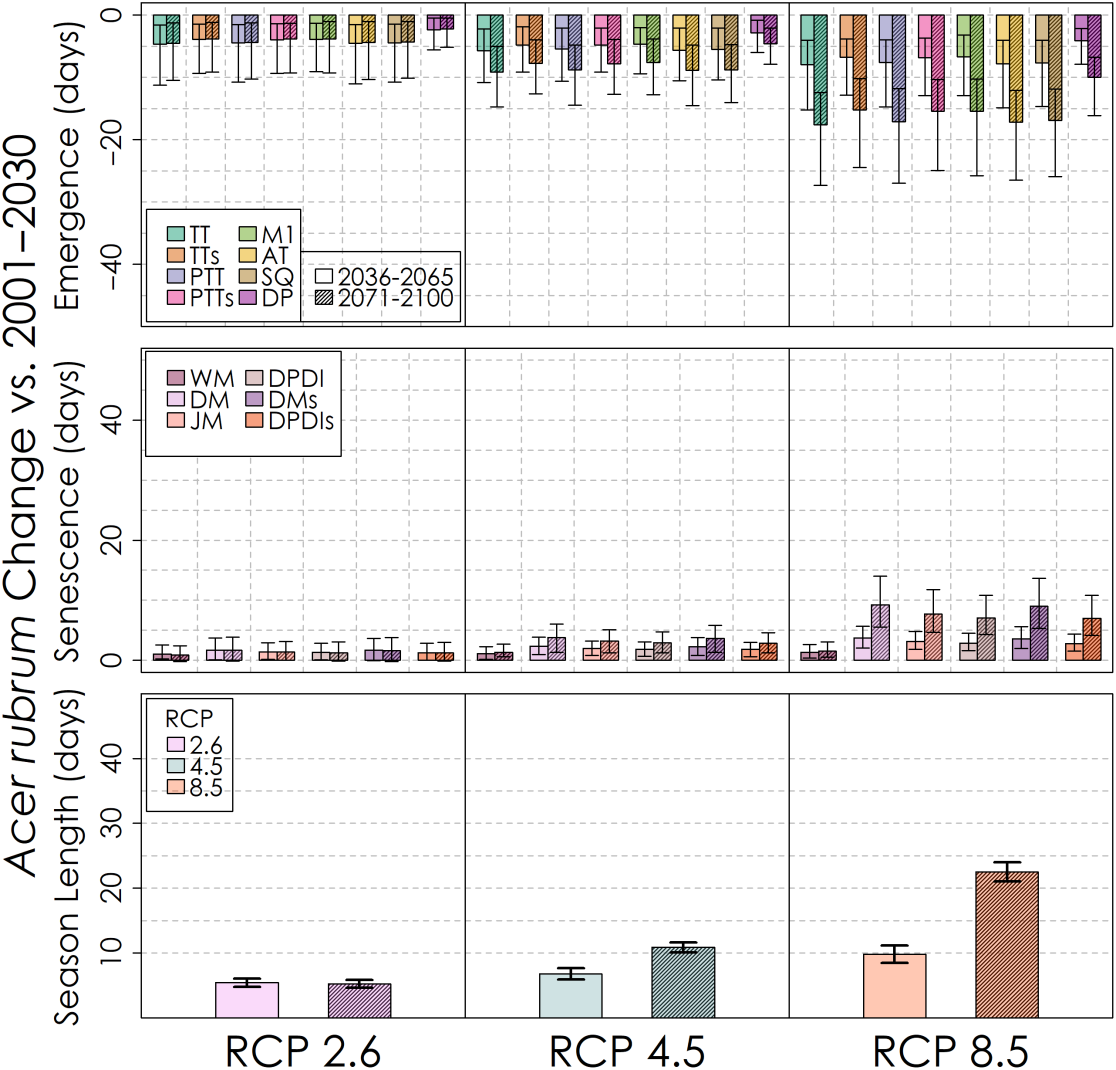
Predicted change in *Acer rubrum* leaf phenology and growing season length for each leaf phenology model and RCP scenario. Uncertainty bars denote the 5th–95th percentile change values.

**FIGURE 14:**
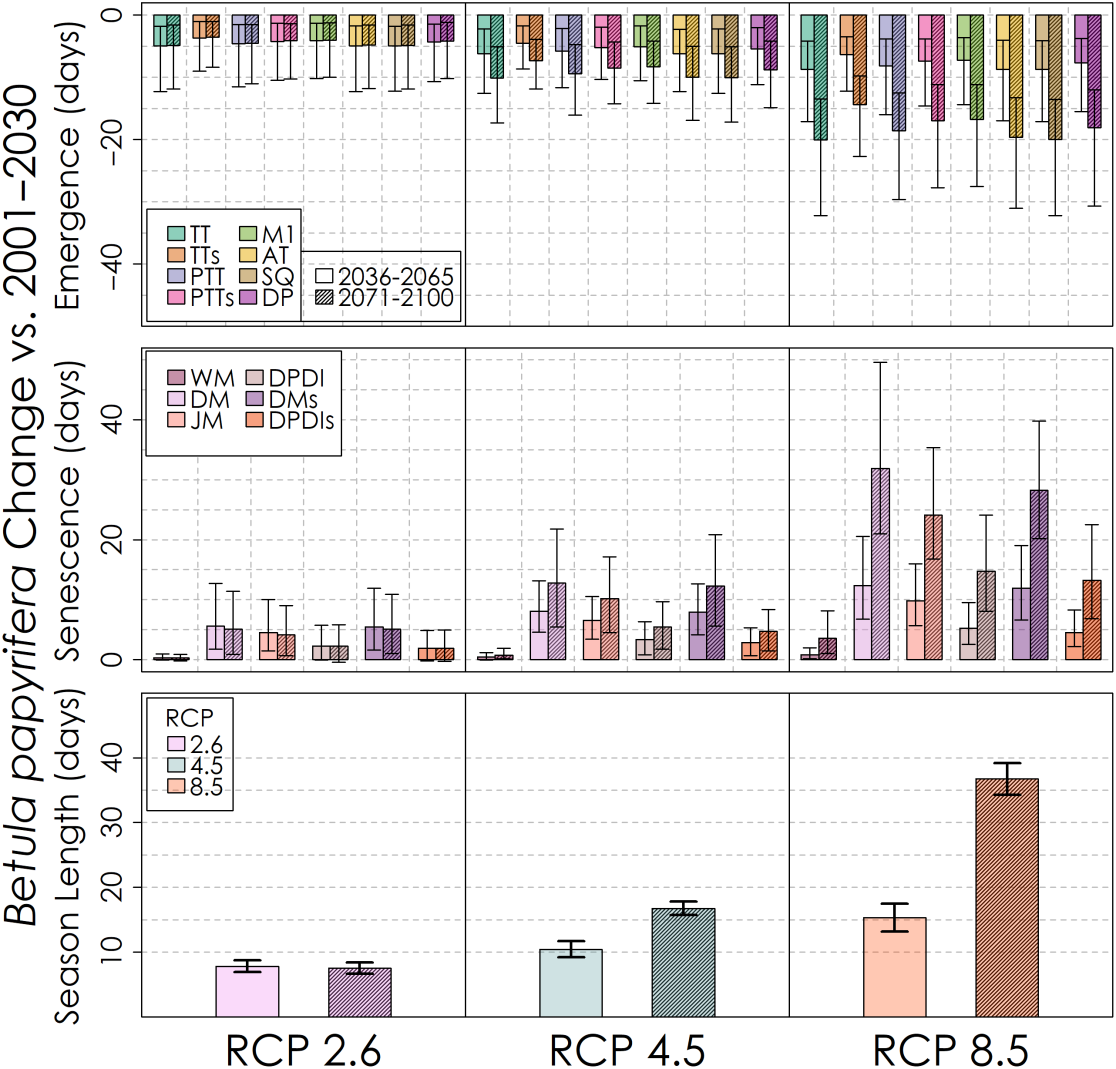
Predicted change in *Betula papyrifera* leaf phenology and growing season length for each leaf phenology model and RCP scenario. Uncertainty bars denote the 5th–95th percentile change values.

**FIGURE 15:**
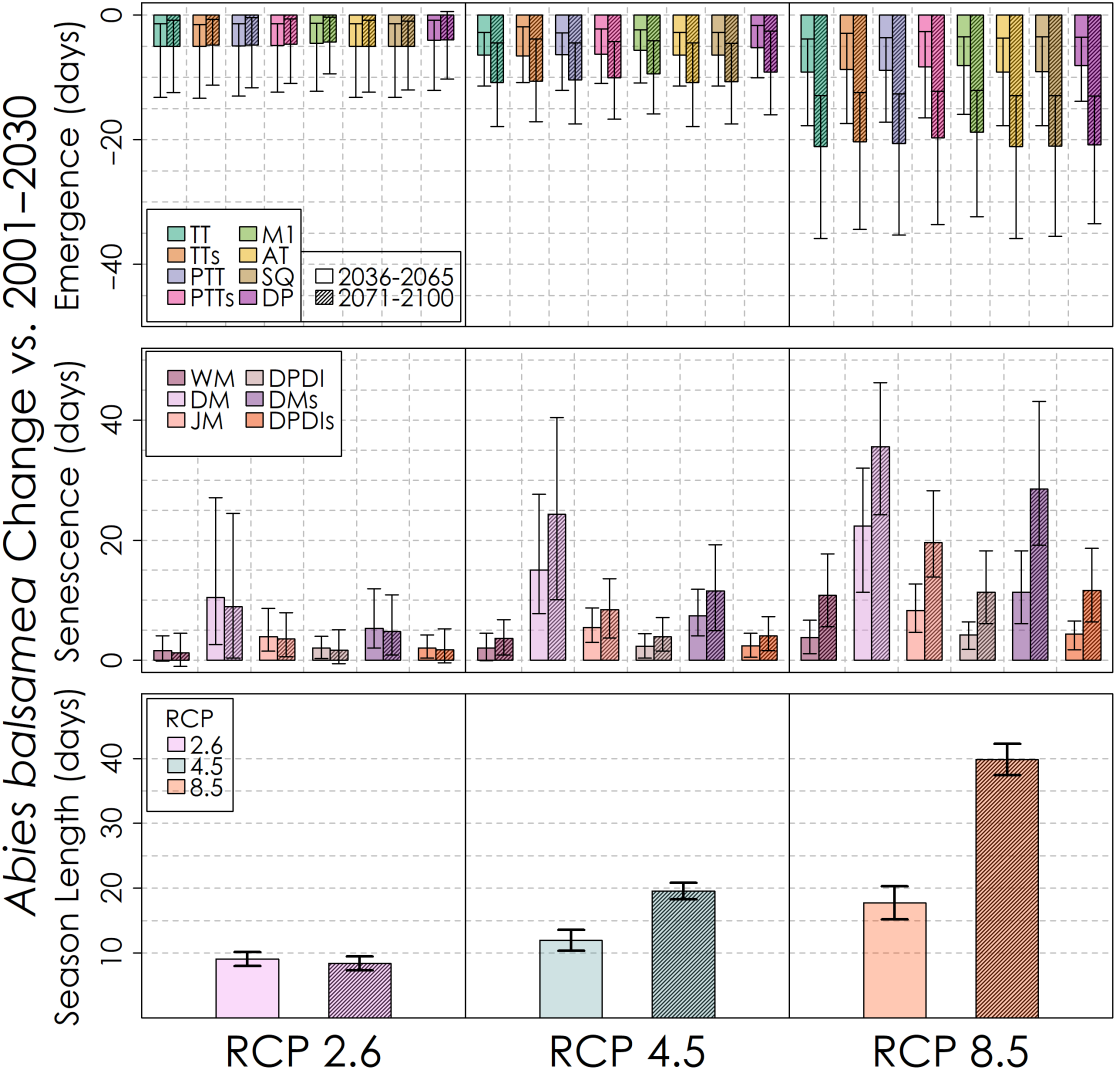
Predicted change in *Abies balsamea* leaf phenology and growing season length for each leaf phenology model and RCP scenario. Uncertainty bars denote the 5th–95th percentile change values.

The projected change in the timing of leaf emergence, leaf senescence and the corresponding season length for the mid‐ and late‐21st century for each phenology model and emissions scenario is shown in Figure 13 for *Acer rubrum*, Figure 14 for *Betula papyrifera* and Figure 15 for *Abies balsamea*. For each species and phenology model, leaf emergence is projected to advance and leaf senescence is projected to be delayed, though by varying degrees among species and models. Interestingly, the counteracting effects of warming on leaf emergence represented in the Dormphot model show a lesser advance relative to other models for *Acer rubrum* under all emissions scenarios. In contrast, the projected delay in leaf senescence increased continuously with both time and emissions in a similar fashion across leaf senescence models, though with the exception of the trigger‐based White model. On average under a high emissions scenario by the end of the 21st century, the length of the growing season is to be extended by about 3 weeks for *Acer rubrum* and five or more weeks for *Betula papyrifera* and *Abies balsamea*, respectively. For each species, the relative extension in season length due to either the earlier leaf emergence or later leaf senescence varies. For *Acer rubrum*, 69% of the extension in season length is due to an earlier leaf emergence (16 days of 22‐day average). For *Betula papyrifera* 49% of the extension in season length is due to an earlier leaf emergence (18 days of 37‐day average). For *Abies balsamea* 51% of the extension in season length is due to an earlier leaf emergence (20 days of 40‐day average). On average, advances in leaf emergence under high emissions by the end of the 21st century were similar across species ranging from 16 to 22 days, while delays in leaf senescence vary from just 7 days for *Acer rubrum* to 19 and 20 days for *Betula papyrifera* and *Abies balsamea*, respectively.

## 4 DISCUSSION

Here, we present novel phenocam observations and predictions of leaf phenology for three tree species of the Acadian Forest Region using species‐specific calibrated process models. An extension of the growing season in the context of warming is likely for all species by the late 21st century under both moderate and high emissions scenarios with a variety of leaf phenology models. The magnitude of this extension varies depending upon species, with a greater extension predicted for more boreal‐climate‐suited species *Betula papyrifera* and *Abies balsamea* versus more temperate‐climate suited *Acer rubrum* due to a more pronounced leaf senescence delay. The species‐specific projections from our models agree with the findings of a 5‐year experimental study by Montgomery et al. (2020) in which species with a higher latitude of origin had a greater response to experimental warming. Given the expected northward expansion of the more temperate‐climate‐suited species like *Acer rubrum*, the species‐specific differences in projected phenology and season length have important implications for carbon uptake and ecological interactions within the Acadian Forest Region (Kharouba et al., 2018; Lafleur et al., 2010).

### 4.1 Model performances

Our study provided a novel demonstration of the parameterization of species‐specific leaf phenology models using phenocam observations for species with broadly distinct biogeographical ranges. Most models showcased similar performance with no singular outstanding model despite the diversity of biogeographical ranges across species, suggesting each model is well suited for the purposes of phenology modelling. This is in agreement with previous studies (Basler, 2016; Hufkens et al., 2018; Liu et al., 2020). Nevertheless, the optimal model varied across validation exercises for each species, suggesting different underlying cue mechanisms for these species despite their cohabitation.

The Dormphot leaf emergence model was originally calibrated with experimentation on *Betula pubescens* and found to be superior to simpler models with datasets from across Europe (Caffarra et al., 2011). The high performance of this model for a variety of training and validation sample configurations we found suggests this model is flexible and transferable to other regions such as the Acadian Forest Region. The suitability of the Dormphot model along with other models which included photoperiod shows that photoperiod likely exerts an important control on the process of leaf emergence for species in the Acadian Forest Region as well as across the PhenoCam Network.

For leaf senescence modelling, incorporating the influence of the preceding spring leaf emergence did not lead to overall improved model performance, though it improved regional applicability for the Acadian Phenocam Network in the case of *Acer rubrum*. Model performance among leaf senescence models was consistent between models relative to leaf emergence. Prediction error among leaf senescence models was approximately twice that of leaf emergence models. The parameterization of several of the leaf senescence models used in this study is mathematically similar to leaf emergence models, despite the distinction in the relationship for each phenophase to growing season temperatures (Figure 4). This indicates there is ample potential for improving leaf senescence models. Future leaf senescence model development would benefit from the exploration of novel cues and parameterizations that are more distinct from leaf emergence models.

The environmental context for both training and validation datasets was highly influential on ultimate model performance. We found variable model performance when models were validated with warm site‐years versus cold site‐years, with different optimal models depending upon each context for both leaf emergence and senescence. Another study using satellite‐based observations and modelling also found model performances varied based on validation temperatures (Fu et al., 2014). Together this performance bias suggests that despite the satisfactory performance of these phenology models, novel parameterizations and potentially additional drivers are needed to improve the models' general applicability to a range of seasonal conditions.

The magnitude of error for each model was generally within the range from previous studies for both leaf emergence (~1 week) and senescence models (~1 to 3 weeks; Basler, 2016; Fang et al., 2022; Liu et al., 2020). Leaf senescence model error found with *Abies balsamea* was relatively high. In a study that also used the PhenoCam Version 2.0 dataset with a simplified version of the Delpierre model (Delpierre et al., 2009) for leaf senescence, Fang et al. (2022) reported RMSE values of ~17 days for evergreen needleleaf forest sites though just ~7 days for deciduous broadleaf forest sites. The higher leaf senescence error observed for evergreen needleleaf species such as *Abies balsamea* in our study and other studies may be due to the greater challenge of obtaining precise leaf phenology observations for evergreen needleleaf species. Evergreen needleleaf species exhibit more subtle and gradual changes in colour than deciduous species, resulting in seasonal greenness curves with reduced amplitudes and subsequently less precise phenology extraction. In addition, the rising portion of the greenness curve for evergreen needleleaf species is the result of both the emergence of new leaves as well as the greening of existing leaves, compounding uncertainty for process model development with phenocam observations (Seyednasrollah et al., 2021). Despite this, the error for leaf emergence estimation with *Abies balsamea* in our study was only slightly higher than the other species while for Fang et al. (2022) RMSE was similarly high for both phenophases for evergreen needleleaf forest sites relative to deciduous broadleaf sites. More work is needed to improve the precision of leaf phenology extraction from phenocam‐derived observations of evergreen needleleaf vegetation. That being said, constraining amplitude thresholds in photosynthetic leaf phenology based on colour changes through phenocams rather than manually observable leaf phenophase changes may allow for broad comparisons between observations of leaf phenology for evergreen species (Seyednasrollah et al., 2021).

Another source of uncertainty influencing both leaf emergence and senescence models may be regionally differing phenological constraints which are not always captured by model equations and parameters, such as with a fixed daylength threshold. For example, Moon et al. (2021) reported a transition in the relative importance of temperature and photoperiod affecting leaf emergence around the 10°C isotherm. The high and sometimes consistent model error for leaf senescence models herein over that of leaf emergence models may be due to each leaf senescence model including only temperature and photoperiod as drivers. Additional phenomena are known to influence the timing of leaf senescence, such as moisture availability, the seasonal timing of warming anomalies, minimum temperatures, frost frequency, consecutive dry days, heat stress, drought, consecutive rainy days and consecutive heavy rain days (Bigler & Vitasse, 2021; Lang et al., 2019; Liu et al., 2020; Xie, Civco, & Silander Jr, 2018; Xie, Wang, et al., 2018; Zohner & Renner, 2019). In addition, there is growing evidence that the probability of frost has an important influence on leaf phenology (Marquis et al., 2020). Incorporating these variables and potentially new variables yet to be discovered may help to improve the performance of both leaf emergence and senescence models. In addition, more observations and modelling studies focused on autumn phenology are needed as there is a deficit of research on leaf senescence relative to leaf emergence (Fang et al., 2022; Gallinat et al., 2015).

For the global combination of samples in our study, complex leaf emergence models representing the combination of dormancy induction, endodormancy and ecodormancy release slightly outperformed more simple models for several validation exercises. In contrast, when we conducted leave‐one‐out and k‐fold cross‐validations, we found that complex models performed less well than simple models such as the M1 model. For the pooled combination of samples within our study, complex leaf senescence models incorporating the influence of the preceding ecodormancy release generally performed less well than simpler models. This may be due to the distinction in season length across regions, such that the parameterization for the constraint of growing season length in one region is less applicable to another region. When we conducted leave‐one‐out and k‐fold cross‐validations for leaf senescence, we again found that complex models performed less well than simple models such as the trigger‐based White model. Together these findings are consistent with Basler (2016) for both global and regional transferability error evaluations in relation to model complexity. This is likely due to the trade‐off between global performance and local specificity with complex models, which achieve greater performance with higher validation sample sizes. Globally trained models tended to predict an earlier date of leaf emergence and a later date of senescence than is observed for the Acadian Forest Region. This suggests that both model complexity and training dataset spatial coverage should be considered when parameterizing, validating and developing models. Additionally, this indicates that relationships between leaf phenology and environmental influences may vary in a non‐linear fashion between regions, calling for more local and regional scale studies to inform broad leaf phenology mechanism understandings.

Despite the broad range of observations and training contexts in our study, all species and validations agree in that simple Thermal Time model is not optimal relative to other models which include additional drivers. The Thermal Time model is often used to simulate leaf phenology within Dynamic Global Vegetation Models that form the terrestrial vegetational component of Land Surface Models within Earth System Models (Arora & Boer, 2005; Cox, 2001). The Thermal Time model is also commonly used to communicate expected changes in the duration of the vegetational growing season (Government of Canada, 2022; https://www.nrcan.gc.ca/climate‐change/climate‐change‐impacts‐forests/forest‐change‐indicators/growing‐season/18470). Model performances in our study indicate that the inclusion of additional drivers for leaf emergence and senescence could improve the realism of dynamic global vegetation models and predictions of vegetation growing season length.

### 4.2 Future leaf phenology for Acadian Forest region

In the Acadian Forest Region, future leaf emergence in the context of both moderate and high emissions will be earlier while leaf senescence will be later, though more work is needed to better predict the magnitude of these changes. The ensemble of models in our study predicts about 2, 2–3 and 3 weeks advance in leaf emergence and 1, 2–3 and 2–3 week(s) delay in leaf senescence by the end of this century with high emissions for *Acer rubrum*, *Betula papyrifera* and *Abies balsamea*, respectively. In projecting future leaf phenology patterns, we found divergent patterns between simple and complex models. Under high emissions in the later century, leaf emergence shows lesser advancement within models including endodormancy release chilling constraints, such as the Dormphot model, relative to other models. For leaf senescence, complex models including the influence of the preceding spring leaf emergence show similar or lesser delays in leaf senescence over time and emissions intensity in comparison to their counterpart models. The complex Dormphot model performed well in warmer years, suggesting it is a valuable tool for projecting leaf emergence in the context of future climate, and that the constrained advancement in leaf emergence it predicts is therefore likely (Caffarra et al., 2011). The reduced advancement in leaf emergence over time predicted with the Dormphot model agrees with the non‐linearity for leaf emergence temperature sensitivity found by Chen et al. (2019) with observations from 1950 to 2013 and for Flynn and Wolkovich (2018) with an experimental study. This suggests that as winter temperatures increase, the advancement in leaf emergence may be constrained by reduced chilling exposure accumulation for each of our species.

Caution is warranted in the interpretation of this apparent diminishing response of leaf emergence phenology to temperature change as more work is needed to thoroughly examine this finding. The aggregated weather patterns from the CMIP5 models may underestimate local interannual variability, omit the important role of anomalous seasonal conditions and underestimate phenological temperature sensitivity due to uncertainty in projected temperatures (Keenan et al., 2020). For leaf senescence during warm site‐years, the trigger‐based White model and the Delpierre model without the preceding spring leaf emergence influence outperformed other models. These models also produced widely divergent predictions of leaf senescence delay with time, the White model predicted a week or less delay across species while the Delpierre model predicted as much as 4 weeks delay. Despite the constrained advance in leaf emergence, it was still greater or similar to the projected delay in leaf senescence across species. This somewhat contrasts with the findings of Fu et al. (2018). With both a cooling and warming treatment for the European species *Fagus sylvatica*, Fu et al. (2018) found a greater temperature sensitivity for leaf senescence than emergence. Together this suggests that different species in different climate regions may exhibit diverse phenological responses to changing temperature regimes. The greater leaf emergence advancement relative to leaf senescence delay was most pronounced for the temperate species *Acer rubrum*, which had only a minor projected leaf senescence delay. Improved leaf senescence models are therefore needed to foster more confident projections of growing season length.

The continuous leaf senescence delay we found with time and emissions intensity agrees with the sustained leaf senescence temperature sensitivity found by Chen et al. (2019) with observations from 1950 to 2013. A study projecting changes in leaf senescence timing by the late 21st century including *Acer rubrum* and *Betula papyrifera* reported a similar delay in the onset of fall colouration for these species, though while using different predictive models which included moisture availability effects (Xie, Wang, et al., 2018). The projected delay in leaf colouration onset therein was about 1 week for *Acer rubrum* and just over 2 weeks for *Betula papyrifera* (Xie, Wang, et al., 2018). While a limited advancement in leaf emergence overtime translates to a potentially limited lengthening in the growing season, the expected continuous delay in leaf senescence may still promote increased seasonal carbon uptake as Wu et al. (2013) found that the timing of leaf senescence was more influential on seasonal carbon uptake than leaf emergence. If alternatively, the timing of leaf senescence depends upon the timing of leaf emergence as some studies have found and our Acadian Network observations suggest (Keenan & Richardson, 2015; Liu et al., 2020), this may constrain the length of growing season and subsequently carbon uptake. Another important consideration is the occurrence of anomalously early leaf emergence timings with respect to adjacent years or the long‐term record, as this makes leaves susceptible to frost damage and may lead to carbon losses (Augspurger, 2009, 2013; Chamberlain et al., 2019; Gu et al., 2008; Montgomery et al., 2020; Richardson, Hufkens, Milliman, Aubrecht, Furze, et al., 2018; Vitasse et al., 2018). In addition, while not represented in our models, climate change has the potential to influence leaf phenology through alternative effects including changes in moisture availability and disturbance legacy effects (Angulo‐Sandoval et al., 2004; Meier et al., 2021; Spafford et al., 2023; Wu et al., 2022). Together this indicates more regional‐scale species‐specific observation and modelling efforts are needed to understand regionally variable controls of leaf senescence as well as leaf emergence.

### 4.3 Future implications

A substantial annual temperature increase of ~4°C is predicted for the Acadian Forest Region under a high emissions scenario. This change in conditions will surpass the optimal growing temperature for boreal‐climate‐suited species like *Abies balsamea* and *Betula papyrifera* (Dhar et al., 2014; Frank, 1990; Wang et al., 1998). Previous studies have predicted a decline in the proportion of boreal species in the Acadian Forest by the late 21st century due to suboptimal growing conditions (Taylor et al., 2017), which is supported by the dramatic shifts predicted with our phenology models for the 21st century. An experimental study by Vaughn et al. (2021) found reduced mortality and sustained height growth in the context of drought for temperate‐climate‐suited *Acer rubrum* in comparison to colder‐climate‐adapted species such as *Abies balsamea*. In a synthesis of the effects of climate change on *Abies balsamea* regeneration, Collier et al. (2022) alternatively found that the adverse impacts on *Abies balsamea* may occur with a complex combination of processes including reduced competitive fitness and mortality of overstory trees. The potentially extensive delay in the timing of leaf senescence found in our study for boreal species in the Acadian Forest Region suggests that future nutrient resorption success may be diminished for these species in comparison to temperate species. Leaf senescence functions primarily as a means of conserving nutrients for deciduous tree species which are used in the development of new leaves in the following spring (Estiarte & Peñuelas, 2015). A delayed senescence leads to a greater risk of nutrient losses due to fall hurricanes prematurely removing or damaging leaf tissues, disrupting the normal course of nutrient recycling achieved through senescence. Together this indicates that more boreal‐typical species like *Abies balsamea* in the Acadian Forest may suffer declined growth, greater mortality, reduced fitness, a shift in optimal biogeographical envelopes beyond their current range and perhaps a substantially reduced longevity in the context of climate change in the 21st century. This has important implications for forest structure and ecological interactions across the Acadian Forest Region, which is already vulnerable due to most species therein being near the limit of their ranges (Fisichelli et al., 2014; Körner et al., 2016; Pearson & D'Orangeville, 2022; Wang et al., 2021).

The predicted lengthening of the carbon uptake period prompted by an earlier leaf emergence and later leaf senescence for each species found in our study may be expected to lead to increased carbon uptake (Wu et al., 2013). On the contrary, increased mortality, disturbance and suboptimal growing conditions in the context of climate change may lead to reduced carbon uptake across the Acadian Forest Region (Taylor et al., 2020), losses which may more than compensate for potential carbon uptake gains from warming (D'Orangeville et al., 2018). In addition, several studies have found that a longer leafing period does not always lead to increased carbon uptake in the form of stable woody biomass (Camarero et al., 2022; Čufar et al., 2015; Delpierre et al., 2017; Dow et al., 2022; Fang et al., 2020; Marchand et al., 2021). Further, the potential for the increased establishment of more temperate‐climate‐suited species like *Acer rubrum* may be limited throughout the 21st century due to the physical occupation of space by boreal species (Taylor et al., 2017). As species ranges shift northward and weather patterns exhibit more frequent and intense anomalies in the context of climate change, there is also a greater potential for carbon losses due to late spring frost events which may be more damaging for species outside their native ranges (Vitasse et al., 2014; Zanne et al., 2018). Therefore, the composition of the Acadian Forest Region is likely to change under a high emissions scenario by the late 21st century, as well as the capacity for carbon uptake within the Acadian Forest Region. Understanding which phenological strategy is optimal in response to such changes is necessary for promoting the migration of suitable species and provenances therein (Ding & Brouard, 2022).

Beyond biogeochemistry, the constrained delay in leaf senescence for temperate species such as *Acer rubrum* found in our study has important implications for the autumn colour ecotourism industry in the Acadian Forest Region (Ivakhiv, 2005; Spencer & Holecek, 2007). *Acer rubrum* are responsible for the vibrant red colours which contrast with the predominant yellow autumn colouration of other species in much of the Acadian Forest Region. Divergence in the relative timing of leaf senescence for deciduous species of the Acadian Forest may have important implications for the future appearance and appeal of the fall colours, as well as for ecological interactions between species (Cleland et al., 2007; Kharouba et al., 2018; Renner & Zohner, 2018). Cleland et al. (2012) found that species that do not respond as acutely to temperature changes may in fact be at a disadvantage in terms of ecological performance relative to other species, despite the better potential avoidance of suboptimal growing conditions with such strategies.

## 5 CONCLUSION

The Acadian Forest Region is a unique transitional zone composed of both boreal and temperate forest species in eastern North America. Leaf phenology, the timing of season leaf life cycle events, responds directly to climate change and thus serves as an important biological indicator. Stationary timelapse cameras, known as phenocams, are cost‐effective monitoring tools that can provide spatially and temporally replicated species‐specific observations of leaf phenology in a range of climatic contexts. We collected four growing seasons of observations for the species *Acer rubrum* (red maple), *Betula papyrifera* (paper/white birch) and *Abies balsamea* (balsam fir) across the Acadian Phenocam Network and accessed multiple growing season observations of these species from the North American PhenoCam Network. With these observations, we conducted species‐specific parameterizations of eight leaf emergence and six leaf senescence models which encompass a range in process and driver representation, resulting in 42 unique leaf phenology models. With these models, we simulated future patterns in leaf emergence, senescence and season length (senescence minus emergence) for these species at sites within the Acadian Phenocam Network based on projected weather from Climate Models within the Fifth Phase of the Coupled Model Intercomparison Project (CMIP5). All models performed similarly well, with model errors in the range observed by previous studies for both leaf emergence and senescence. Leaf emergence was better predicted by more complex models while leaf senescence was better predicted by relatively simple models. By the end of the 21st century with moderate or high emissions, leaf emergence will likely be 2 weeks earlier while the magnitude of leaf senescence change varies across species and models. Temperate species like *Acer rubrum* may have as little as a 1‐week delay in leaf senescence while leaf senescence for boreal species like *Betula papyrifera* and *Abies balsamea* may be 2 to 4 weeks later. Consequently, the length of growing season extension varies from about 3 weeks for *Acer rubrum* to more than 5 weeks for *Betula papyrifera* and *Abies balsamea*. This differential response pattern between boreal and temperate species in the Acadian Forest Region has important implications for forest ecology as well as biogeochemistry and forest‐based sectors of the economy (e.g. forestry, ecotourism). A promising avenue to enhance the confidence of leaf phenology predictions in the context of climate change is the improved monitoring and modelling of leaf senescence. Our work demonstrates phenocams have the potential to rapidly advance process‐based model development and, therefore, foster more confident predictions of leaf phenology.

## AUTHOR CONTRIBUTIONS

**Lynsay Spafford:** Conceptualization (equal); data curation (lead); formal analysis (lead); investigation (lead); methodology (equal); resources (supporting); software (lead); validation (lead); visualization (lead); writing – original draft (lead); writing – review and editing (equal). **Andrew MacDougall:** Conceptualization (equal); data curation (supporting); formal analysis (supporting); funding acquisition (lead); investigation (supporting); methodology (equal); resources (lead); software (supporting); supervision (lead); validation (supporting); visualization (supporting); writing – original draft (supporting); writing – review and editing (equal). **James Steenberg:** Conceptualization (equal); data curation (supporting); formal analysis (supporting); funding acquisition (supporting); investigation (supporting); methodology (equal); resources (supporting); software (supporting); validation (supporting); visualization (supporting); writing – original draft (supporting); writing – review and editing (equal).

## Data Availability

Data used in this study are available in Section A2 in Appendix A and the PhenoCam V2.0 dataset (Seyednasrollah, Young, Hufkens, Milliman, Friedl, Frolking, Richardson, Abraha, et al., 2019; https://daac.ornl.gov/VEGETATION/guides/PhenoCam_V2.html).

## APPENDIX A

### A1 LEAF EMERGENCE AND SENESCENCE MODEL EQUATIONS, OPTIMAL PARAMETERS, AND PARAMETERIZATION RANGES

**TABLE A1:** Leaf emergence process models included in this study for estimating the timing of the start of season (SOS) or leaf emergence. Note that each leaf emergence site‐year runs from September 1st to August 31st such that a starting date of January 1st corresponds to a *t*_0_ of 103. *R*_*frc*_ and *R*_*chl*_ denote the rate of forcing and chilling, respectively. *S*_*frc*_ and *S*_*chl*_ denote the accumulated state of forcing and chilling, respectively. *DR*_*t*_ and *DR*_*p*_ denote the rate of dormancy induction based on temperature and photoperiod, respectively. *S*_DR_ denotes the state of dormancy induction accumulation.

Model	Variables, parameters, and *release process(es) included*	Equation	Reference
Null	‐ Mean date from all observations ${\bar{\mathrm{SOS}}}_{o}$ ***None***	$\mathrm{SOS}={\bar{\mathrm{SOS}}}_{o}$	—
Thermal Time (TT)	‐ Starting date *t*_0_ ‐ Daily mean temperature *T*_*i*_ ‐ Base temperature for accumulation *T*_*b*_ ‐ Critical threshold for leaf emergence *F*_*crit*_ ***Ecodormancy***	$R_{\mathrm{frc}}\left(i\right)=\left\{\begin{array}{c} T_{i}>T_{b}:T_{i}-T_{b}\\ T_{i}\leq T_{b}:\;0\; \end{array}\right.$ $S_{\mathrm{frc}}=\sum_{i=t_{0}}^{n}R_{\mathrm{frc}}$ $S_{\mathrm{frc}}\geq F_{\text{crit}}$	Hufkens et al. (2018), Basler (2016), Wang (1960), Réaumur (1735)
Thermal Time with Sigmoidal Temperature Response (TTs)	‐ Starting date *t*_0_ ‐ Daily mean temperature *T*_*i*_ ‐ Response parameter *b* ‐ Response parameter *c* ‐ Critical threshold for leaf emergence *F*_*crit*_ ***Ecodormancy***	$R_{\mathrm{frc}}\left(i\right)=\frac{1}{1+e^{-b\left(T_{i}-c\right)}}$ $S_{\mathrm{frc}}=\sum_{i=t_{0}}^{n}R_{\mathrm{frc}}$ $S_{\mathrm{frc}}\geq F_{\text{crit}}$	Hufkens et al. (2018), Basler (2016), Kramer (1994), Hänninen (1990)
Photo‐Thermal Time (PTT)	‐ Starting date *t*_0_ ‐ Daily mean temperature *T*_*i*_ ‐ Base temperature for accumulation *T*_*b*_ ‐ Daylength *L*_*i*_ ‐ Critical threshold for leaf emergence *F*_*crit*_ ***Ecodormancy***	$R_{\mathrm{frc}}\left(i\right)=\;\frac{L_{i}}{24}*\left\{\begin{array}{c} T_{i}>T_{b}:\;T_{i}-T_{b}\\ T_{i}\leq T_{b}:\;0\; \end{array}\right.$ $S_{\mathrm{frc}}=\sum_{i=t_{0}}^{n}R_{\mathrm{frc}}$ $S_{\mathrm{frc}}\geq F_{\text{crit}}$	Hufkens et al. (2018), Basler (2016), Črepinšek et al. (2006), Masle (1989)
Photo‐Thermal Time with Sigmoidal Temperature Response (PTTs)	‐ Starting date *t*_0_ ‐ Daily mean temperature *T*_*i*_ ‐ Response parameter *b* ‐ Response parameter *c* ‐ Daylength *L*_*i*_ ‐ Critical threshold for leaf emergence *F*_*crit*_ ***Ecodormancy***	$R_{\mathrm{frc}}\left(i\right)=\frac{L_{i}}{24}*\frac{1}{1+e^{-b\left(T_{i}-c\right)}}$ $S_{\mathrm{frc}}=\sum_{i=t_{0}}^{n}R_{\mathrm{frc}}$ $S_{\mathrm{frc}}\geq F_{\text{crit}}$	Hufkens et al. (2018), Basler (2016), Črepinšek et al. (2006), Kramer (1994), Hänninen (1990), Masle (1989)
M1	‐ Starting date *t*_0_ ‐ Daily mean temperature *T*_*i*_ ‐ Base temperature for accumulation *T*_*b*_ ‐ Daylength *L*_*i*_ ‐ Response parameter *k* ‐ Critical threshold for leaf emergence *F*_*crit*_ ***Ecodormancy***	$R_{\mathrm{frc}}\left(i\right)={\left(\frac{L_{i}}{10}\right)}^{k}*\;\left\{\begin{array}{c} T_{i}>T_{b}:T_{i}-T_{b}\\ T_{i}\leq T_{b}:\;0\; \end{array}\right.$ $S_{\mathrm{frc}}=\sum_{i=t_{0}}^{n}R_{\mathrm{frc}}$ $S_{\mathrm{frc}}\geq F_{\text{crit}}$	Hufkens et al. (2018), Basler (2016), Blümel and Chmielewski (2012)
Alternating (AT)	‐ Starting date *t*_0_ ‐ Daily mean temperature *T*_*i*_ ‐ Base temperature for accumulation *T*_*b*_ ‐ Response parameter *a* ‐ Response parameter *b* ‐ Response parameter *c* ***Endodormancy & Ecodormancy***	$R_{\mathrm{chl}}\left(i\right)=\left\{\begin{array}{c} T_{i}<T_{b}:\;0\\ T_{i}\geq T_{b}:\;1 \end{array}\right.$ $S_{\mathrm{chl}}=\sum_{i=t_{0}}^{n}R_{\mathrm{chl}}$ $R_{\mathrm{frc}}\left(i\right)=\left\{\begin{array}{c} T_{i}>T_{b}:T_{i}-T_{b}\\ T_{i}\leq T_{b}:\;0\; \end{array}\right.$ $S_{\mathrm{frc}}=\sum_{i=t_{0}}^{n}R_{\mathrm{frc}}$ $F_{\text{crit}}=a+b*e^{c*S_{\mathrm{chl}}}$ $S_{\mathrm{frc}}\geq F_{\text{crit}}$	Hufkens et al. (2018), Basler (2016), Murray et al. (1989), Cannell and Smith (1983)
Sequential (SQ)	‐ Chilling starting date *t*_0*c*_ ‐ Forcing starting date *t*_0*f*_ ‐ Daily mean temperature *T*_*i*_ ‐ Minimum temperature for chilling accumulation *T*_*min*_ ‐ Optimal temperature for chilling accumulation *T*_*opt*_ ‐ Maximum temperature for chilling accumulation *T*_*max*_ ‐ Threshold for forcing accumulation *C*_*req*_ ‐ Base temperature for forcing accumulation *T*_*b*_ ‐ Critical threshold for leaf emergence *F*_*crit*_ ***Endodormancy & Ecodormancy***	$R_{\text{tchl}}\left(i\right)=\left\{\begin{array}{c} T_{\min}\leq T_{i}<T_{\mathrm{opt}}:\;\frac{T_{i}-T_{\min}}{T_{\mathrm{opt}}-T_{\min}\;}\;\\ T_{\mathrm{opt}}\leq T_{i}<T_{\max}:1-\frac{T_{i}-T_{\mathrm{opt}}}{T_{\max}-T_{\mathrm{opt}}\;}\\ T_{i}<T_{\min}:0\;\\ T_{i}>T_{\max}:0\; \end{array}\right.$ $S_{\mathrm{chl}}=\sum_{i=t_{0c}}^{n}R_{\text{tchl}}$ $k=\left\{\begin{array}{c} S_{\mathrm{chl}}<C_{\mathrm{req}}:0\\ S_{\mathrm{chl}}\geq C_{\mathrm{req}}:1 \end{array}\right.$ $R_{\mathrm{frc}}\left(i\right)=k*\left\{\begin{array}{c} T_{i}>T_{b}:T_{i}-T_{b}\\ T_{i}\leq T_{b}:\;0\; \end{array}\right.$ $S_{\mathrm{frc}}=\sum_{i=t_{0f}}^{n}R_{\mathrm{frc}}$ $S_{\mathrm{frc}}\geq F_{\text{crit}}$	Hufkens et al. (2018), Basler (2016), Kramer (1994), Hänninen (1990)
Dormphot (DP)	‐ Dormancy induction sensitivity parameter *a* ‐ Daily mean temperature *T*_*i*_ ‐ Response parameter for dormancy induction accumulation *b* ‐ Daylength *L*_*i*_ ‐ Threshold daylength for dormancy induction accumulation *L*_*crit*_ ‐ Threshold for dormancy induction *D*_*crit*_ ‐ Chilling sensitivity parameter *c* ‐ Rate of chilling accumulation parameter *d* ‐ Forcing sensitivity parameter *h*_*L*_ ‐ Chilling threshold parameter for forcing accumulation *C*_*req*_ ‐ Daylength sensitivity parameter *gT* ‐ Forcing accumulation parameter *df* ‐ Critical threshold for leaf emergence *F*_*crit*_ ***Dormancy Induction, Endodormancy, & Ecodormancy***	${\mathrm{DR}}_{t}\left(i\right)=\frac{1}{1+e^{a\left(\;T_{i}-b\right)}}$ ${\mathrm{DR}}_{p}\left(i\right)=\frac{1}{1+e^{10\left(\;L_{i}-L_{\text{crit}}\right)}}$ $S_{\mathrm{DR}}=\sum_{i=t_{0}}^{n}{\mathrm{DR}}_{t}*{\mathrm{DR}}_{p}$ $R_{\mathrm{chl}}\left(i\right)=\left\{\begin{array}{c} S_{\mathrm{DR}}<D_{\text{crit}}:\;0\\ S_{\mathrm{DR}}\geq D_{\text{crit}}:\frac{1}{1+e^{c{\left(\;T_{i}-d\right)}^{2}+\left(\;T_{i}-d\right)}} \end{array}\right.$ $S_{\mathrm{chl}}=\sum_{i=t_{0}}^{n}R_{\mathrm{chl}}$ ${\mathrm{dl}}_{50}\left(i\right)=\frac{24}{1+e^{h_{L}\left(S_{\mathrm{chl}}\left(i\right)-C_{\mathrm{req}}\right)}}$ $T_{50}\left(i\right)=\frac{60}{1+e^{\mathrm{gT}\left(L_{i}-{\mathrm{dl}}_{50}\right)}}$ $R_{\mathrm{frc}}\left(i\right)=\left\{\begin{array}{c} S_{\mathrm{DR}}<D_{\text{crit}}:\;0\\ S_{\mathrm{DR}}\geq D_{\text{crit}}:\frac{1}{1+e^{\mathrm{df}\left(\;T_{i}-T_{50}\left(i\right)\;\right)}} \end{array}\right.$ $S_{\mathrm{frc}}=\sum_{i=t_{0}}^{n}R_{\mathrm{frc}}$ $S_{\mathrm{frc}}\geq F_{\text{crit}}$	Hufkens et al. (2018), Basler (2016), Caffarra et al. (2011)

**TABLE A2:** Optimal parameters and initial parameter range in square brackets for or each leaf emergence model using general simulated annealing. Note that each leaf emergence site‐year runs from September 1st to August 31st such that a starting date of January 1st corresponds to a *t*_0_ of 103.

Model	Parameters	*Acer rubrum*	*Betula papyrifera*	*Abies balsamea*
Thermal Time (TT)	‐ Starting date *t*_0_ ‐ Base temperature for accumulation *T*_*b*_ ‐ Critical threshold for leaf emergence *F*_*crit*_	*t*_0_: 192 [1–365] *T*_*b*_: 4.062 [−5 to +10] *F*_*crit*_: 212.5 [0 to 2000]	*t*_0_: 181 [1–365] *T*_*b*_: 4.804 [−5 to +10] *F*_*crit*_: 157.6 [0 to 2000]	*t*_0_: 175 [1–365] *T*_*b*_: 9.989 [−5 to +10] *F*_*crit*_: 6.584 [0 to 2000]
Thermal Time with Sigmoidal Temperature Response (TTs)	‐ Starting date *t*_0_ ‐ Response parameter *b* ‐ Response parameter *c* ‐ Critical threshold for leaf emergence *F*_*crit*_	*t*_0_: 185 [1–365] *b*: 0.1443 [0 to 100] *c*: 30.74 [0 to 100] *F*_*crit*_: 2.188 [0 to 350]	*t*_0_: 193 [1–365] *b*: 0.1778 [0 to 100] *c*: 20.33 [0 to 100] *F*_*crit*_: 5.147 [0 to 350]	*t*_0_: 177 [1–365] *b*: 2.1472 [0 to 100] *c*: 12.99 [0 to 100] *F*_*crit*_: 0.9415 [0 to 350]
Photo‐Thermal Time (PTT)	‐ Starting date *t*_0_ ‐ Base temperature for accumulation *T*_*b*_ ‐ Critical threshold for leaf emergence *F*_*crit*_	*t*_0_: 191 [1–365] *T*_*b*_: 4.888 [−5 to +10] *F*_*crit*_: 103.7 [0 to 2000]	*t*_0_: 181 [1–365] *T*_*b*_: 4.319 [−5 to +10] *F*_*crit*_: 101.8 [0 to 2000]	*t*_0_: 173 [1–365] *T*_*b*_: 9.918 [−5 to +10] *F*_*crit*_: 3.876 [0 to 2000]
Photo‐Thermal Time with Sigmoidal Temperature Response (PTTs)	‐ Starting date *t*_0_ ‐ Response parameter *b* ‐ Response parameter *c* ‐ Critical threshold for leaf emergence *F*_*crit*_	*t*_0_: 185 [1–365] *b*: 0.1696 [0 to 100] *c*: 30.36 [0 to 100] *F*_*crit*_: 0.8392 [0 to 350]	*t*_0_: 177 [1–365] *b*: 0.2260 [0 to 100] *c*: 16.94 [0 to 100] *F*_*crit*_: 4.019 [0 to 350]	*t*_0_: 178 [1–365] *b*: 2.552 [0 to 100] *c*: 12.79 [0 to 100] *F*_*crit*_: 0.6102 [0 to 350]
M1	‐ Starting date *t*_0_ ‐ Base temperature for accumulation *T*_*b*_ ‐ Response parameter *k* ‐ Critical threshold for leaf emergence *F*_*crit*_	*t*_0_: 151 [1–365] *T*_*b*_: 6.708 [−5 to +10] *k*: 4.991 [0 to 5] *F*_*crit*_: 710.7 [0 to 2000]	*t*_0_: 174 [1–365] *T*_*b*_: 4.753 [−5 to +10] *k*: 3.079 [0 to 5] *F*_*crit*_: 470.1 [0 to 2000]	*t*_0_: 174 [1–365] *T*_*b*_: 9.996 [−5 to +10] *k*: 4.392 [0 to 5] *F*_*crit*_: 31.98 [0 to 2000]
Alternating (AT)	‐ Starting date *t*_0_ ‐ Base temperature for accumulation *T*_*b*_ ‐ Response parameter *a* ‐ Response parameter *b* ‐ Response parameter *c*	*t*_0_: 193 [1–365] *T*_*b*_: 3.425 [−5 to +10] *a*: 226.2 [0 to 500] *b*: 2.549 [0 to 1000] *c*: 4.774 [0 to 5]	*t*_0_: 182 [1–365] *T*_*b*_: 4.023 [−5 to +10] *a*: 36.65 [0 to 500] *b*: 148.1 [0 to 1000] *c*: 0.7998 [0 to 5]	*t*_0_: 175 [1–365] *T*_*b*_: 9.999 [−5 to +10] *a*: 0.8163 [0 to 500] *b*: 5.783 [0 to 1000] *c*: 2.780 [0 to 5]
Sequential (SQ)	‐ Chilling starting date *t*_0*c*_ ‐ Forcing starting date *t*_0*f*_ ‐ Minimum temperature for chilling accumulation *T*_*min*_ ‐ Optimal temperature for chilling accumulation *T*_*opt*_ ‐ Maximum temperature for chilling accumulation *T*_*max*_ ‐ Threshold for forcing accumulation *C*_*req*_ ‐ Base temperature for forcing accumulation *T*_*b*_ ‐ Critical threshold for leaf emergence *F*_*crit*_	*t*_0*c*_: 30 [1–365] *t*_0*f*_: 192 [1–365] *T*_*min*_: −3.191 [−5 to +10] *T*_*opt*_: 7.873 [−5 to +10] *T*_*max*_: 14.56 [−5 to +10] *C*_*req*_: 12.82 [0 to 350] *T*_*b*_: 3.009 [−5 to +10] *F*_*crit*_: 246.8 [0 to 2000]	*t*_0*c*_: 14 [1–365] *t*_0*f*_: 181 [1–365] *T*_*min*_: −3.882 [−5 to +10] *T*_*opt*_: 0.3103 [−5 to +10] *T*_*max*_: 1.314 [−5 to +10] *C*_*req*_: 2.801 [0 to 350] *T*_*b*_: 4.816 [−5 to +10] *F*_*crit*_: 157.2 [0 to 2000]	*t*_0*c*_: 19 [1–365] *t*_0*f*_: 177 [1–365] *T*_*min*_: −4.331 [−5 to +10] *T*_*opt*_: 5.636 [−5 to +10] *T*_*max*_: 12.01 [−5 to +10] *C*_*req*_: 3.838 [0 to 350] *T*_*b*_: 9.984 [−5 to +10] *F*_*crit*_: 6.641 [0 to 2000]
Dormphot (DP)	‐ Dormancy induction sensitivity parameter *a* ‐ Response parameter for dormancy induction accumulation *b* ‐ Threshold daylength for dormancy induction accumulation *L*_*crit*_ ‐ Threshold for dormancy induction *D*_*crit*_ ‐ Chilling sensitivity parameter *c* ‐ Rate of chilling accumulation parameter *d* ‐ Forcing sensitivity parameter *h*_*L*_ ‐ Chilling threshold parameter for forcing accumulation *C*_*req*_ ‐ Daylength sensitivity parameter *gT* ‐ Forcing accumulation parameter *df* ‐ Critical threshold for leaf emergence *F*_*crit*_	*a*: 4.351 [−5 to +5] *b*: 13.95 [0 to 100] *L*_*crit*_: 11.11 [8 to 14] *D*_*crit*_: 35.89 [0 to 100] *c*: 0.3875 [0 to 5] *d*: 17.88 [0 to 100] *h*_*L*_: 0.0383 [0 to 20] *C*_*req*_: 0.7199 [0 to 100] *gT*: 0.6398 [0 to 10] *df*: −0.3400 [−1000 to 0] *F*_*crit*_: 10.34 [0 to 100]	*a*: 2.775 [−5 to +5] *b*: 18.25 [0 to 100] *L*_*crit*_: 12.54 [8 to 14] *D*_*crit*_: 21.89 [0 to 100] *c*: 0.01198 [0 to 5] *d*: 79.89 [0 to 100] *h*_*L*_: 16.14 [0 to 20] *C*_*req*_: 27.51 [0 to 100] *gT*: 0.1176 [0 to 10] *df*: −0.5319 [−1000 to 0] *F*_*crit*_: 16.04 [0 to 100]	*a*: 1.807 [−5 to +5] *b*: 3.461 [0 to 100] *L*_*crit*_: 13.16 [8 to 14] *D*_*crit*_: 10.42 [0 to 100] *c*: 0.6685 [0 to 5] *d*: 16.39 [0 to 100] *h*_*L*_: 2.321 [0 to 20] *C*_*req*_: 0.2033 [0 to 100] *gT*: 4.6868 [0 to 10] *df*: −386.4 [−1000 to 0] *F*_*crit*_: 4.005 [0 to 100]

**TABLE A3:** Leaf senescence process models included in this study. Note that each leaf senescence site‐year runs from January 1st to December 31st such that a starting date of January 1st corresponds to a *t*_0_ of 1. *R*_*tp*_, *R*_*t*_, and *R*_*p*_ denote the rate of temperature cooling and photoperiod reducing, the rate of temperature cooling, and the rate of photoperiod reducing, respectively. *S*_*tp*_, *S*_*t*_, and *S*_*p*_ denote the accumulated state of temperature cooling and photoperiod reducing, the accumulated state of temperature cooling, and the accumulated state of photoperiod reducing, respectively.

Model	Variables, parameters, and *process(es) included*	Equation	Reference
Null	‐ Mean date from all observations ${\bar{\mathrm{EOS}}}_{o}$ ***None***	$\mathrm{EOS}={\bar{\mathrm{EOS}}}_{o}$	—
White (WM)	‐ Starting date fixed to July 1st ‐ Daily mean temperature $T_{i}$ ‐ Temperature for combined temperature daylength senescence trigger $T_{b1}$ ‐ Daylength $L_{i}$ ‐ Daylength for combined temperature daylength senescence trigger $L_{\text{crit}}$ ‐ Temperature for singular temperature senescence trigger $T_{b2}$ ‐ Criteria for senescence: first day of either $R_{\mathrm{tp}}$ or $R_{t}$ not equal to 0 ***Dormancy Induction***	Rtpi=Ti<Tb1∧Li<Lcrit:1Ti≥Tb1∨Li≥Lcrit:0 Rti=Ti<Tb2:1Ti≥Tb2:0 $R_{\mathrm{tp}}>0$ $R_{t}>0$	Liu et al. (2020), White et al. (1997)
Delpierre (DM)	‐ Starting date fixed to July 1st ‐ Daily mean temperature $T_{i}$ ‐ Daylength $L_{i}$ ‐ Temperature threshold and parameter for accumulation $T_{b}$ ‐ Daylength threshold and parameter for accumulation $L_{\text{crit}}$ ‐ Exponential weight parameter for temperature importance x ‐ Exponential weight parameter for daylength importance y ‐ Critical threshold for leaf senescence $F_{\text{crit}}$ ***Dormancy Induction***	Rtpi=Ti<Tb∧Li<Lcrit:Tb−Tix*LiLcrityTi≥Tb∨Li≥Lcrit:0 $S_{\mathrm{tp}}=\sum_{i=182}^{n}R_{\mathrm{tp}}$ $S_{\mathrm{tp}}\geq F_{\text{crit}}$	Liu et al. (2020), Delpierre et al. (2009)
Jeong (JM)	‐ Starting date fixed to July 1st ‐ Daily mean temperature $T_{i}$ ‐ Daylength $L_{i}$ ‐ Temperature threshold and parameter for accumulation $T_{b}$ ‐ Daylength threshold for accumulation $L_{\text{crit}}$ ‐ Critical threshold for leaf senescence $F_{\text{crit}}$ ***Dormancy Induction***	Rtpi=Ti<Tb∧Li<Lcrit:Tb−TiTi≥Tb∨Li≥Lcrit:0 $S_{\mathrm{tp}}=\sum_{i=182}^{n}R_{\mathrm{tp}}$ $S_{\mathrm{tp}}\geq F_{\text{crit}}$	Liu et al. (2020), Jeong and Medvigy (2014)
Dormphot with just Dormancy Induction (DPDI)	‐ Dormancy induction sensitivity parameter a ‐ Daily mean temperature $T_{i}$ ‐ Response parameter for dormancy induction accumulation b ‐ Daylength $L_{i}$ ‐ Threshold daylength for dormancy induction accumulation $L_{\text{crit}}$ ‐ Critical threshold for senescence $D_{\text{crit}}$ ***Dormancy Induction***	$R_{t}\left(i\right)=\frac{1}{1+e^{a\left(\;T_{i}-b\right)}}$ $R_{p}\left(i\right)=\frac{1}{1+e^{10\left(\;L_{i}-L_{\text{crit}}\right)}}$ $S_{\mathrm{tp}}=\sum_{i=182}^{n}R_{t}*R_{p}$ $S_{\mathrm{tp}}\geq D_{\text{crit}}$	Liu et al. (2020); Hufkens et al. (2018); Caffarra et al. (2011)
Delpierre's with Preceding Spring Leaf Emergence (DMs)	‐ Starting date fixed to July 1st ‐ Daily mean temperature $T_{i}$ ‐ Daylength $L_{i}$ ‐ Temperature threshold and parameter for accumulation $T_{b}$ ‐ Daylength threshold and parameter for accumulation $L_{\text{crit}}$ ‐ Exponential weight parameter for temperature importance x ‐ Exponential weight parameter for daylength importance y ‐ Threshold modification parameter of anomaly in leaf emergence in the preceding spring relative to the 30‐year average estimated with the PTTs model $S_{a}$ ‐ Threshold modification parameter a ‐ Threshold modification parameter b ‐ Critical threshold for leaf senescence $F_{\text{crit}}$ ***Preceding Ecodormancy Release & Dormancy Induction***	Rtpi=Ti<Tb∧Li<Lcrit:Tb−Tix*LiLcrityTi≥Tb∨Li≥Lcrit:0 $S_{\mathrm{tp}}=\sum_{i=182}^{n}R_{\mathrm{tp}}$ $S_{a}=\text{SOSy}-{\bar{\mathrm{SOS}}}_{30y}$ $F_{\text{crit}}=a+b*S_{a}$ $S_{\mathrm{tp}}\geq F_{\text{crit}}$	Liu et al. (2020); Delpierre et al. (2009)
Dormphot Dormancy Induction with Preceding Spring Leaf Emergence (DPDIs)	‐ Dormancy induction sensitivity parameter a1 ‐ Daily mean temperature $T_{i}$ ‐ Response parameter for dormancy induction accumulation b1 ‐ Daylength $L_{i}$ ‐ Threshold daylength for dormancy induction accumulation $L_{\text{crit}}$ ‐ Threshold modification parameter of anomaly in leaf emergence in the preceding spring relative to the 30‐year average estimated with the PTTs model $S_{a}$ ‐ Threshold modification parameter a2 ‐ Threshold modification parameter b2 ‐ Critical threshold for leaf senescence $D_{\text{crit}}$ ***Preceding Ecodormancy Release & Dormancy Induction***	$R_{t}\left(i\right)=\frac{1}{1+e^{a1\left(\;T_{i}-b1\right)}}$ $R_{p}\left(i\right)=\frac{1}{1+e^{10\left(\;L_{i}-L_{\text{crit}}\right)}}$ $S_{\mathrm{tp}}=\sum_{i=182}^{n}R_{t}*R_{p}$ $S_{a}=\text{SOSy}-{\bar{\mathrm{SOS}}}_{30y}$ $D_{\text{crit}}=a2+b2*S_{a}$ $S_{\mathrm{tp}}\geq D_{\text{crit}}$	Liu et al. (2020); Caffarra et al. (2011)

**TABLE A4:** Optimal parameters and initial parameter range in square brackets for or each leaf senescence model using general simulated annealing. Note that each leaf senescence site‐year runs from January 1st to December 31st such that a starting date of January 1st corresponds to a $t_{0}$ of 1.

Model	Parameters	*Acer rubrum*	*Betula papyrifera*	*Abies balsamea*
White (WM)	‐ Temperature for combined temperature daylength senescence trigger $T_{b1}$ ‐ Daylength for combined temperature daylength senescence trigger $L_{\text{crit}}$ ‐ Temperature for singular temperature senescence trigger $T_{b2}$	$T_{b1}:$ 28.52 [0 to 30] $L_{\text{crit}}:$ 11.55 [6 to 18] $T_{b2}:$ 7.170 [−20 to +20]	$T_{b1}:$ 16.39 [0 to 30] $L_{\text{crit}}:$ 12.14 [6 to 18] $T_{b2}:$ 3.281 [−20 to +20]	$T_{b1}:$ 10.73 [0 to 30] $L_{\text{crit}}:$ 11.47 [6 to 18] $T_{b2}:$ −1.388 [−20 to +20]
Delpierre (DM)	‐ Temperature threshold and parameter for accumulation $T_{b}$ ‐ Daylength threshold and parameter for accumulation $L_{\text{crit}}$ ‐ Exponential weight parameter for temperature importance x ‐ Exponential weight parameter for daylength importance y ‐ Critical threshold for leaf senescence $F_{\text{crit}}$	$T_{b}:$ 39.76 [0 to 40] $L_{\text{crit}}:$ 12. 92 [6 to 18] x: 2 {0,1,2} y: 0 {0,1,2} $F_{\text{crit}}:$ 16192 [0 to 100,000]	$T_{b}:$ 33.06 [0 to 40] $L_{\text{crit}}:$ 15.33 [6 to 18] x: 1 {0,1,2} y: 2 {0,1,2} $F_{\text{crit}}:$ 981.9 [0 to 100,000]	$T_{b}:$ 22.57 [0 to 40] $L_{\text{crit}}:$ 15.48 [6 to 18] x: 0 {0,1,2} y: 2 {0,1,2} $F_{\text{crit}}:$ 69.73 [0 to 100,000]
Jeong (JM)	‐ Temperature threshold and parameter for accumulation $T_{b}$ ‐ Daylength threshold for accumulation $L_{\text{crit}}$ ‐ Critical threshold for leaf senescence $F_{\text{crit}}$	$T_{b}:$ 34.63 [0 to 50] $L_{\text{crit}}:$ 13.15 [6 to 18] $F_{\text{crit}}:$ 613.1 [0 to 2000]	$T_{b}:$ 30.60 [0 to 50] $L_{\text{crit}}:$ 15.31 [6 to 18] $F_{\text{crit}}:$ 1003 [0 to 2000]	$T_{b}:$ 35.69 [0 to 50] $L_{\text{crit}}:$ 15.48 [6 to 18] $F_{\text{crit}}:$ 1911 [0 to 2000]
Dormphot with just Dormancy Induction (DPDI)	‐ Sensitivity parameter a ‐ Response parameter for accumulation b ‐ Threshold daylength for dormancy induction accumulation $L_{\text{crit}}$ ‐ Threshold for dormancy induction $D_{\text{crit}}$	a: 0.1432 [−5 to +5] b: 2.120 [0 to 100] $L_{\text{crit}}:$ 12.83 [8 to 18] $D_{\text{crit}}:$ 44.42 [0 to 100]	a: 3.141 [−5 to +5] b: 16.31 [0 to 100] $L_{\text{crit}}:$ 12.93 [8 to 18] $D_{\text{crit}}:$ 98.61 [0 to 100]	a: 4.885 [−5 to +5] b: 12.02 [0 to 100] $L_{\text{crit}}:$ 11.56 [8 to 18] $D_{\text{crit}}:$ 33.87 [0 to 100]
Delpierre's with Preceding Spring Leaf Emergence (DMs)	‐ Temperature threshold and parameter for accumulation $T_{b}$ ‐ Daylength threshold and parameter for accumulation $L_{\text{crit}}$ ‐ Exponential weight parameter for temperature importance x ‐ Exponential weight parameter for daylength importance y ‐ Threshold modification parameter a ‐ Threshold modification parameter b	$T_{b}:$ 39.63 [0 to 40] $L_{\text{crit}}:$ 12.88 [6 to 18] x: 2 {0,1,2} y: 0 {0,1,2} a: 15754 [0 to 100,000] b: 0.07018 [0 to 1000]	$T_{b}:$ 39.44 [0 to 40] $L_{\text{crit}}:$ 15.33 [6 to 18] x: 2 {0,1,2} y: 1 {0,1,2} a: 34388 [0 to 100,000] b: 92.98 [0 to 1000]	$T_{b}:$ 39.59 [0 to 40] $L_{\text{crit}}:$ 15.48 [6 to 18] x: 1 {0,1,2} y: 2 {0,1,2} a: 1723 [0 to 100,000] b: 0.05690 [0 to 1000]
Dormphot Dormancy Induction with Preceding Spring Leaf Emergence (DPDIs)	‐ Dormancy induction sensitivity parameter a1 ‐ Response parameter for dormancy induction accumulation b1 ‐ Threshold daylength for dormancy induction accumulation $L_{\text{crit}}$ ‐ Threshold modification parameter a2 ‐ Threshold modification parameter b2	a1: 0.1573 [−5 to +5] b1: 6.478 [0 to 100] $L_{\text{crit}}:$ 12.79 [8 to 18] a2: 63.93 [0 to 100] b2: 0.003743 [0 to 10]	a1: 4.537 [−5 to +5] b1: 16.75 [0 to 100] $L_{\text{crit}}:$ 12.86 [8 to 18] a2: 99.78 [0 to 100] b2: 0.01281 [0 to 10]	a1: 4.701 [−5 to +5] b1: 12.03 [0 to 100] $L_{\text{crit}}:$ 11.59 [8 to 18] a2: 34.37 [0 to 100] b2: 0.001405 [0 to 10]

### A2 LEAF EMERGENCE AND SENESCENCE OBSERVATIONS FOR THE ACADIAN PHENOCAM NETWORK

**TABLE A5:** Leaf emergence observations at sites across the Acadian Phenocam Network used in this study. Day of year (DOY) is the calendar day of year from December 31st of the previous year.

Site	Year	Species	Emergence (DOY)
NE1	2019	*Acer rubrum*	161
NE3	2019	*Acer rubrum*	165
NE4	2019	*Acer rubrum*	162
NE6	2019	*Acer rubrum*	159
SW1	2019	*Acer rubrum*	159
SW2	2019	*Acer rubrum*	164
SW4	2019	*Acer rubrum*	163
SW5	2019	*Acer rubrum*	160
NE4	2020	*Acer rubrum*	156
NE6	2020	*Acer rubrum*	151
SW4	2020	*Acer rubrum*	152
SW5	2020	*Acer rubrum*	150
NE1	2021	*Acer rubrum*	152
NE2	2021	*Acer rubrum*	152
NE3	2021	*Acer rubrum*	151
NE4	2021	*Acer rubrum*	153
NE5	2021	*Acer rubrum*	152
NE6	2021	*Acer rubrum*	145
SW1	2021	*Acer rubrum*	146
SW2	2021	*Acer rubrum*	150
SW3	2021	*Acer rubrum*	147
SW4	2021	*Acer rubrum*	146
SW6	2021	*Acer rubrum*	150
NE1	2022	*Acer rubrum*	152
NE2	2022	*Acer rubrum*	157
NE3	2022	*Acer rubrum*	149
NE4	2022	*Acer rubrum*	160
NE5	2022	*Acer rubrum*	156
NE6	2022	*Acer rubrum*	146
SW1	2022	*Acer rubrum*	145
SW2	2022	*Acer rubrum*	152
SW3	2022	*Acer rubrum*	150
SW4	2022	*Acer rubrum*	147
SW6	2022	*Acer rubrum*	152
NE1	2019	*Betula papyrifera*	158
SW3	2019	*Betula papyrifera*	154
SW4	2019	*Betula papyrifera*	158
SW5	2019	*Betula papyrifera*	155
NE4	2020	*Betula papyrifera*	155
SW4	2020	*Betula papyrifera*	155
SW5	2020	*Betula papyrifera*	145
NE1	2021	*Betula papyrifera*	145
NE2	2021	*Betula papyrifera*	149
NE4	2021	*Betula papyrifera*	148
SW2	2021	*Betula papyrifera*	158
SW4	2021	*Betula papyrifera*	144
SW5	2021	*Betula papyrifera*	142
NE1	2022	*Betula papyrifera*	145
NE2	2022	*Betula papyrifera*	154
NE4	2022	*Betula papyrifera*	153
SW2	2022	*Betula papyrifera*	153
SW3	2022	*Betula papyrifera*	141
SW4	2022	*Betula papyrifera*	147
SW5	2022	*Betula papyrifera*	141
NE1	2019	*Abies balsamea*	160
NE3	2019	*Abies balsamea*	155
NE4	2019	*Abies balsamea*	163
NE6	2019	*Abies balsamea*	158
SW3	2019	*Abies balsamea*	154
SW4	2019	*Abies balsamea*	157
SW6	2019	*Abies balsamea*	147
NE4	2020	*Abies balsamea*	154
NE6	2020	*Abies balsamea*	148
SW4	2020	*Abies balsamea*	147
NE1	2021	*Abies balsamea*	149
NE2	2021	*Abies balsamea*	158
NE3	2021	*Abies balsamea*	144
NE4	2021	*Abies balsamea*	150
NE5	2021	*Abies balsamea*	152
NE6	2021	*Abies balsamea*	146
SW3	2021	*Abies balsamea*	143
SW4	2021	*Abies balsamea*	146
SW6	2021	*Abies balsamea*	158
NE1	2022	*Abies balsamea*	144
NE2	2022	*Abies balsamea*	155
NE3	2022	*Abies balsamea*	142
NE4	2022	*Abies balsamea*	154
NE5	2022	*Abies balsamea*	159
NE6	2022	*Abies balsamea*	144
SW3	2022	*Abies balsamea*	148
SW4	2022	*Abies balsamea*	147
SW6	2022	*Abies balsamea*	158

**TABLE A6:** Leaf senescence observations at sites across the Acadian Phenocam Network used in this study. Day of year (DOY) is the calendar day of year from December 31st of the previous year.

Site	Year	Species	Senescence (DOY)
NE1	2019	*Acer rubrum*	241
NE3	2019	*Acer rubrum*	244
NE4	2019	*Acer rubrum*	298
NE5	2019	*Acer rubrum*	253
NE6	2019	*Acer rubrum*	258
SW1	2019	*Acer rubrum*	263
SW2	2019	*Acer rubrum*	270
SW4	2019	*Acer rubrum*	266
SW5	2019	*Acer rubrum*	256
SW5	2020	*Acer rubrum*	281
SW6	2020	*Acer rubrum*	268
NE1	2021	*Acer rubrum*	270
NE2	2021	*Acer rubrum*	252
NE3	2021	*Acer rubrum*	258
NE4	2021	*Acer rubrum*	243
NE5	2021	*Acer rubrum*	279
NE6	2021	*Acer rubrum*	256
SW1	2021	*Acer rubrum*	271
SW2	2021	*Acer rubrum*	270
SW3	2021	*Acer rubrum*	242
SW4	2021	*Acer rubrum*	237
SW6	2021	*Acer rubrum*	253
NE1	2019	*Betula papyrifera*	241
SW3	2019	*Betula papyrifera*	233
SW4	2019	*Betula papyrifera*	259
SW5	2019	*Betula papyrifera*	275
SW5	2020	*Betula papyrifera*	277
SW6	2020	*Betula papyrifera*	278
NE1	2021	*Betula papyrifera*	224
NE2	2021	*Betula papyrifera*	245
NE4	2021	*Betula papyrifera*	268
SW2	2021	*Betula papyrifera*	248
SW3	2021	*Betula papyrifera*	224
SW4	2021	*Betula papyrifera*	224
SW5	2021	*Betula papyrifera*	286
SW6	2021	*Betula papyrifera*	246
NE1	2019	*Abies balsamea*	249
NE3	2019	*Abies balsamea*	289
NE5	2019	*Abies balsamea*	249
NE6	2019	*Abies balsamea*	259
SW3	2019	*Abies balsamea*	285
SW4	2019	*Abies balsamea*	254
NE1	2021	*Abies balsamea*	264
NE2	2021	*Abies balsamea*	251
NE3	2021	*Abies balsamea*	244
NE4	2021	*Abies balsamea*	273
NE5	2021	*Abies balsamea*	302
NE6	2021	*Abies balsamea*	247
SW3	2021	*Abies balsamea*	286
SW4	2021	*Abies balsamea*	219
SW6	2021	*Abies balsamea*	288
